# ElectroMap: High-throughput open-source software for analysis and mapping of cardiac electrophysiology

**DOI:** 10.1038/s41598-018-38263-2

**Published:** 2019-02-04

**Authors:** Christopher O’Shea, Andrew P. Holmes, Ting Y. Yu, James Winter, Simon P. Wells, Joao Correia, Bastiaan J. Boukens, Joris R. De Groot, Gavin S. Chu, Xin Li, G. Andre Ng, Paulus Kirchhof, Larissa Fabritz, Kashif Rajpoot, Davor Pavlovic

**Affiliations:** 1 Institute of Cardiovascular Sciences, University of Birmingham, Birmingham, UK; 2 EPSRC Centre for Doctoral Training in Physical Sciences for Health, School of Chemistry, University of Birmingham, Birmingham, UK; 3 School of Computer Science, University of Birmingham, Birmingham, UK; 4 Institute of Clinical Sciences, University of Birmingham, Birmingham, UK; 5 Institute of Microbiology and Infection, School of Biosciences, University of Birmingham, Birmingham, UK; 6 Amsterdam UMC, University of Amsterdam, Department of Anatomy and Physiology, Amsterdam, The Netherlands; 7 Amsterdam UMC, University of Amsterdam, Heart Center, Department of Cardiology, Amsterdam, The Netherlands; 8 Department of Cardiovascular Sciences, University of Leicester, Leicester, UK; 9 NIHR Leicester Biomedical Research Centre, Glenfield Hospital, Leicester, UK; 10 Department of Cardiology, UHB NHS Trust, Birmingham, UK; 11 Department of Physiology, University of Melbourne, Melbourne, Australia

## Abstract

The ability to record and analyse electrical behaviour across the heart using optical and electrode mapping has revolutionised cardiac research. However, wider uptake of these technologies is constrained by the lack of multi-functional and robustly characterised analysis and mapping software. We present ElectroMap, an adaptable, high-throughput, open-source software for processing, analysis and mapping of complex electrophysiology datasets from diverse experimental models and acquisition modalities. Key innovation is development of standalone module for quantification of conduction velocity, employing multiple methodologies, currently not widely available to researchers. ElectroMap has also been designed to support multiple methodologies for accurate calculation of activation, repolarisation, arrhythmia detection, calcium handling and beat-to-beat heterogeneity. ElectroMap implements automated signal segmentation, ensemble averaging and integrates optogenetic approaches. Here we employ ElectroMap for analysis, mapping and detection of pro-arrhythmic phenomena in silico, in cellulo, animal model and *in vivo* patient datasets. We anticipate that ElectroMap will accelerate innovative cardiac research and enhance the uptake, application and interpretation of mapping technologies leading to novel approaches for arrhythmia prevention.

## Introduction

The incidence and prevalence of cardiac diseases continues to increase every year^1,2^. Adequate prevention and treatment requires a better understanding of the mechanistic drivers^3^. Detailed understanding of spatial and temporal electrical behaviour and ionic handling across the heart is integral to this.

Ability to measure field potentials and conduction from multiple sites can be achieved by contact/non-contact electrodes arrays. More recently, optical mapping has allowed measurement and mapping of action potential and calcium transient morphology and conduction, leading to a broader utilisation of mapping technologies in the cardiovascular sphere. Increasingly, electrode array mapping has made its way from pre-clinical to clinical arena whilst the use of optical mapping continues to expand in experimental research. Optically imaging intact tissue using voltage or calcium sensitive fluorescent dyes has advantages over surface electrodes but the requirement for contraction uncouplers and inability to perform *in vivo* experiments limits clinical utility of optical mapping^4^. Nevertheless, insights from optical mapping experiments have informed our understanding of complex arrhythmias and electrophysiological remodelling in heart disease^5–8^. Electrode mapping has equally provided vital knowledge^9,10^, demonstrating that re-entry drives ventricular tachycardia^11^, actively guiding current clinical ablation strategies. Some arrhythmogenic principles, for example in atrial fibrillation (AF), remain poorly understood from a mechanistic standpoint^12,13^ and thus require further experimentation.

Increased availability of optical mapping hardware in the laboratory has led to expansion of this technology. Further uptake and wider application is hindered by limitations with respect to data processing and analysis. This challenge is intensified as developing camera technology provides ever-increasing spatio-temporal resolution. Furthermore, multiple processing algorithms are employed before the underlying data can be interpreted^14,15^. These algorithms require computational expertise to implement and are commonly developed and used within individual research groups, utilising techniques specific to camera resolution, file type and animal species. Whilst some software solutions are accessible, even straightforward calculation of conduction velocity (CV) across the heart is currently unavailable, but to a few specialist groups. There is an unmet need for a robustly tested mapping software that allows high-throughput data processing, analysis, and mapping of electrophysiology from different acquisition modalities and diverse datasets with distinct electrophysiological (EP) properties (for example: animal, human tissue and cell monolayers). Therefore, we present novel and robust open-source software, ElectroMap, for analysis of voltage and calcium optical mapping data. This work builds upon our previously published algorithms^15–17^ while integrating analytic approaches developed and validated by others^6,14,18,19^. ElectroMap provides analysis of key EP parameters including action potential and calcium transient morphology, calcium decay constant (τ), activation and repolarisation times, diastolic interval (DI), time-to-peak, phase mapping and dominant frequency (DF). A key innovation is the introduction of a comprehensive CV module for robust investigation of CV changes. This module integrates established single vector^18^ and multi vector^20^ techniques for CV measurement as well as a novel “activation constant” analysis. Furthermore, semi-automated alternans analysis is enabled through development of a comprehensive alternans detection and quantification module. ElectroMap integrates automated pacing frequency recognition, ensemble (i.e. multi-beat) averaging and beat-to-beat analysis options. Here we employ ElectroMap for analysis, mapping and detection of pro-arrhythmic phenomena in silico, in cellulo, animal model and *in vivo* patient datasets, and thus demonstrate its utility for cardiac research.

## Methods

Expanded details about methods are provided in the Supplementary Material. All animal procedures were undertaken in accordance with ethical guidelines set out by the UK Animals (Scientific Procedures) Act 1986 and Directive 2010/63/EU of the European Parliament on the protection of animals used for scientific purposes. Studies conformed to the Guide for the Care and Use of Laboratory Animals published by the U.S. National Institutes of Health under assurance number A5634-01. Experiments were approved by the home office (mouse: PPL 30/2967 and PFDAAF77F, guinea pig: PPL PF75E5F7F) and the institutional review boards at University of Birmingham (murine) and King’s College London (guinea pig). For optical mapping of human tissue, left atrial appendages were obtained from patients with atrial fibrillation (AF) during thoracoscopic surgery, as described before^21^. The study was in accordance with the declaration of Helsinki and approved by the Review Board of the Academic Medical Center, Amsterdam. *In vivo* intracardiac data were obtained in a clinical study approved by local ethics committee for patients undergoing AF ablation at the University Hospitals of Leicester NHS Trust as previously described^22^. All patients gave written informed consent.

### Optical Mapping

Both mouse and guinea pig optical mapping experiments were conducted as previously reported. For experiments in mouse^7,15,17^ fluorescent dye (Voltage: DI-4-ANEPPS, Calcium: Rhod-2AM) was loaded into Langendorff perfused whole hearts. Left atria were isolated, with the posterior atrial surface exposed. Atria were superfused under normoxic (95%O_2_/5%CO_2_) or hypoxic (95%N_2_/5%CO_2_) conditions. Pacing was performed via bipolar platinum electrode, and fluorescence captured using ORCA flash 4.0 camera (1 kHz sampling rate, maximum field of view: 200 × 2048 pixels, 71 µm/pixel, Hamamatsu Electronics, Japan). Isolated guinea pig hearts^8^ were loaded with voltage dye Di-8-ANEPPS, paced via silver bipolar electrodes and imaged using Evolve Delta 512 × 512 pixel EMCCD cameras (500 Hz sampling rate, 64 × 64 pixels, 320 µm/pixel, Photometrics, USA) mounted on a Olympus MVX10 stereomicroscope.

Human left atrial appendages^21^ were removed using an endoscopic stapling device (Endo Gia stapler, Tyco Healthcare Group) and transported to the optical mapping setup in 100 mL cooled superfusion fluid. Atrial preparation was submerged in a recording chamber, loaded with Di-4-ANEPPS and paced using an epicardial electrode. MiCAM Ultima camera (2 kHz sampling rate, 100 × 100pixels, 100 µm/pixel SciMedia, USA) was used to record epicardial images.

### *In-vivo* Human Mapping

A high-density non-contact multi-electrode array catheter (Ensite Array, St. Jude Medical, USA) was positioned in the right atrium. Virtual unipolar electrograms were bandpass filtered from 1–100 Hz with additional noise filtering, then exported from a 2048 node geometry as previously described^22^.

### ElectroMap Design and Development

**Figure 1:**
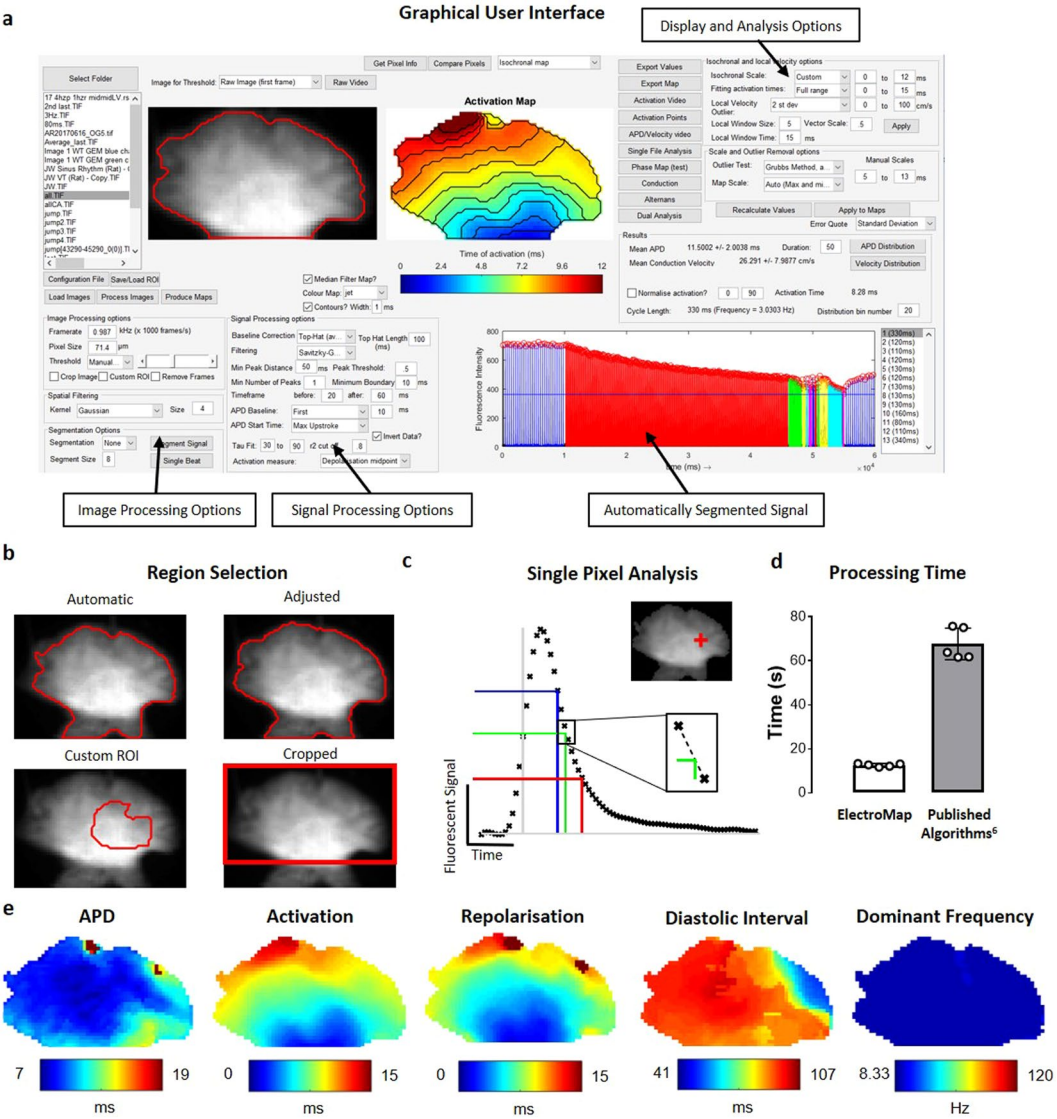
ElectroMap interface and function. (**a**) ElectroMap Graphical User Interface with automatic signal segmentation, image processing, signal processing, display and analysis options highlighted. (**b**) Illustration of 4 different region selection methods available in ElectroMap. (**c**) Example of analysis from one pixel (top inset) within murine atrial images. Action potential duration (APD) 30 (blue), 50 (green) and 70 (red) are calculated from time of maximum upstroke velocity (vertical grey line) to 30%, 50%, 70% repolarisation respectively. Time of repolarisation calculated by linear interpolation (dotted line) between sampling points, as shown for 50% repolarisation in lower inset. (**d**) Time taken to process and analyse 5 image stacks of murine left atria from raw images to conduction velocity calculation, comparison of ElectroMap and our previously published algorithms. (**e**) Selection of pseudo-colour maps denoting some of the electrophysiological parameters measurable using ElectroMap.

**Figure 2:**
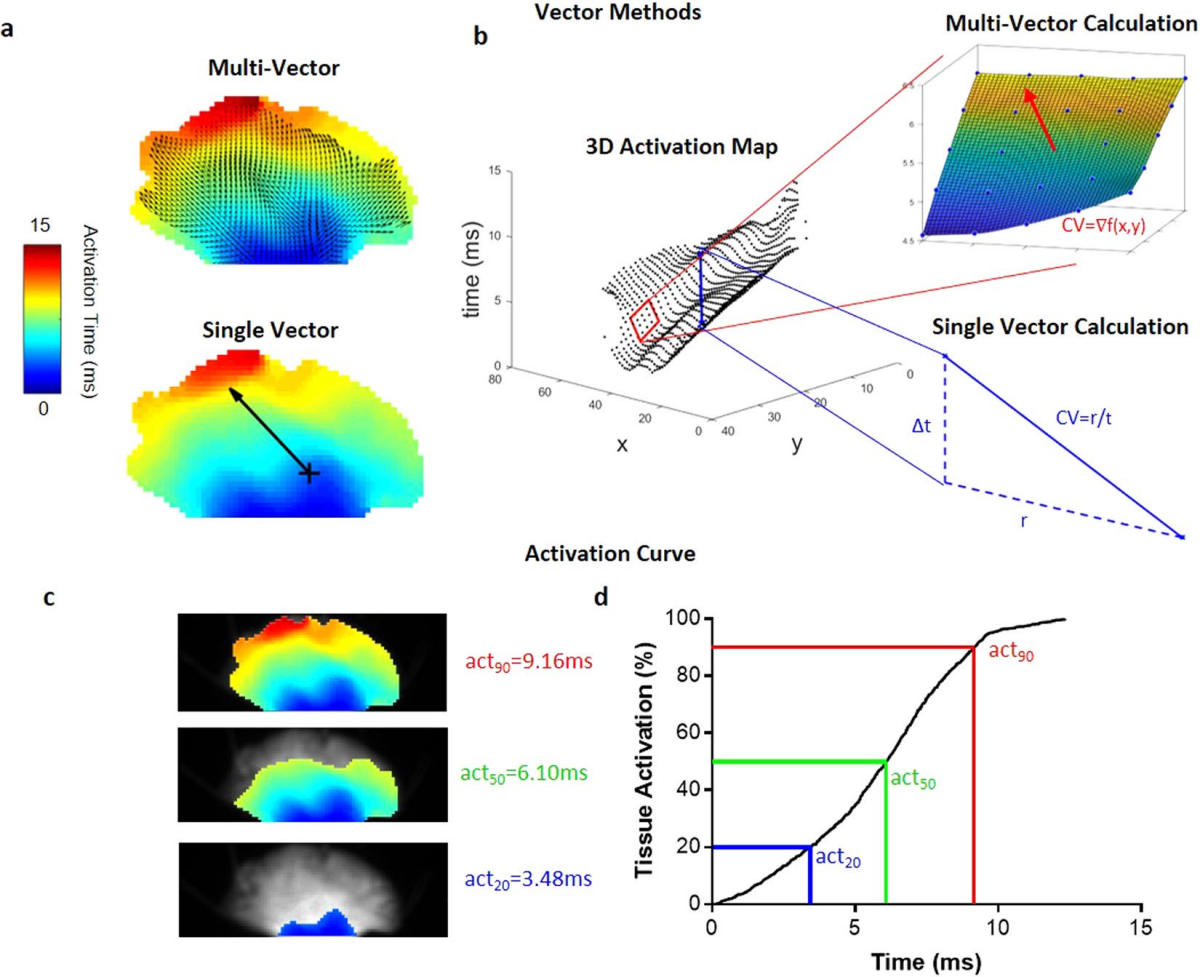
ElectroMap conduction velocity module quantification methods. (**a**) Vector methods applied to murine atria. Top panel: multi-vector method, with local conduction vectors (arrows) calculated across the tissue. Lower panel: single velocity method, where conduction speed is measured along the vector (arrow) connecting two points. (**b**) Activation map represented in 3D with x,y pixel positions and activation time on z-axis. Upper inset demonstrates calculation of one local vector from a 5 × 5 pixel region by fitting of a polynomial surface (f(x,y)) to measured activation times (t(x,y)). Lower inset shows calculation of single vector velocity from activation time difference (Δt) and distance (r) between two selected points. (**c**) Activation of the murine atria at 20% (act_20_), 50% (act_50_), and 90% (act_90_), total activation. (**d**) The associated activation curve, with act_20_, act_50_ and act_90_ highlighted.

**Figure 3:**
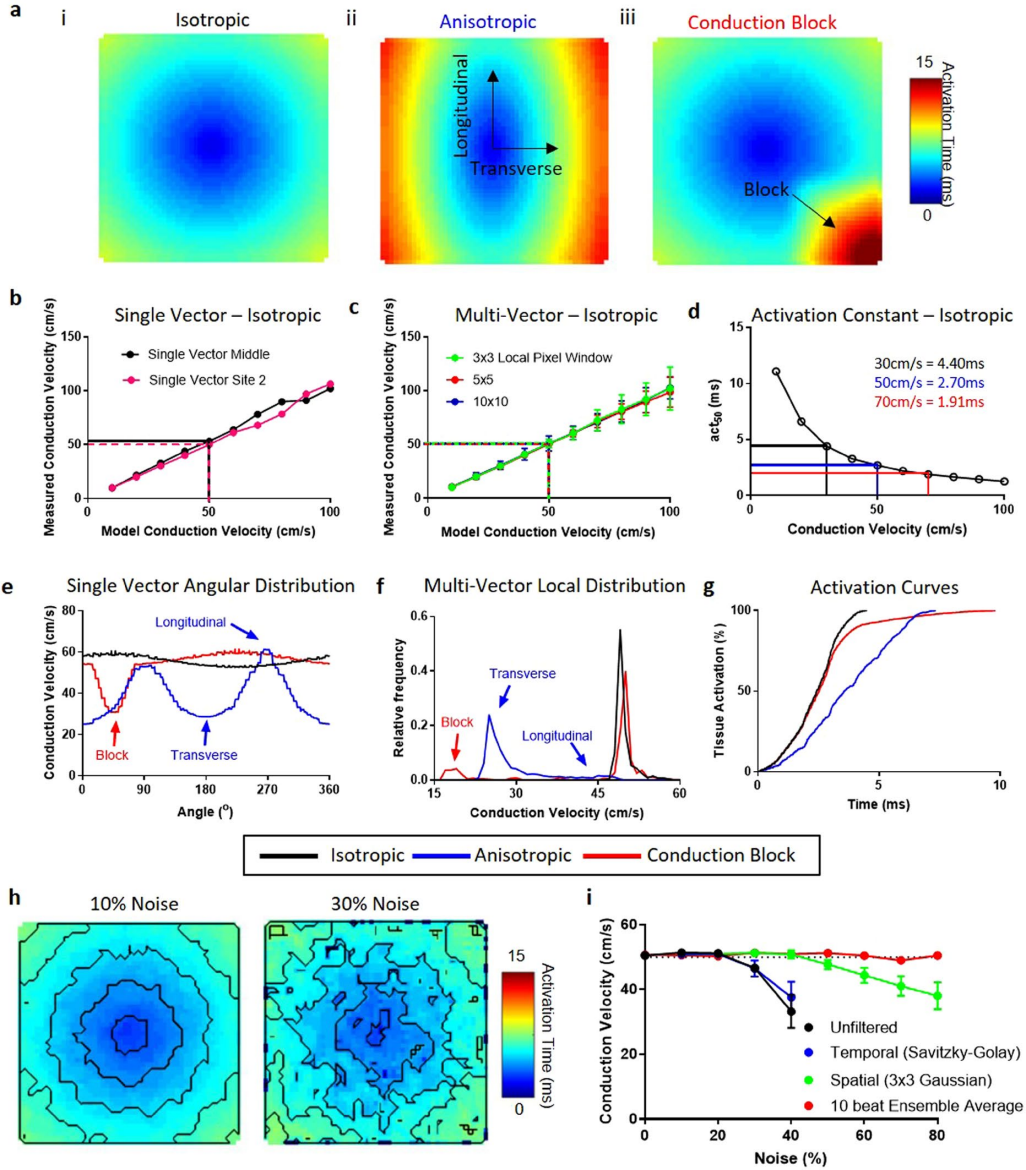
Validation of conduction velocity quantification methods using model datasets. (**a**) Model conduction datasets exhibiting isotropic conduction (i), anisotropic conduction (ii) and isotropic conduction with a 2:1 conduction block in lower right region (iii). (**b**–**d**) Measured conduction velocities (CV) from isotropic conduction model datasets spanning 10 to 100 cm/s using ElectroMap. (**b**) CV is measured using a single vector from centre of sample (black) and from edge of sample (pink). (**c**) multi-vector method is used with windows sizes of 3 × 3 (green), 5 × 5 (red) and 10 × 10 (blue) pixels. CV is then measured as mean of the magnitude of all local vectors calculated, with error bars representing the standard deviation of the mean. (**d**) Activation curve method with act_50_ (time to 50% activation) used to quantify conduction. (**e**–**g**) Three CV quantification methods applied to model datasets with isotropic, anisotropic and conduction block present conduction. In all three cases, fastest model conduction was 50 cm/s. (**e**) Change in measured CV as a function of angle using single vector method from centre of tissue. (**f**) Distribution of local vector magnitude measured by multi-vector method (5 × 5 pixel window) in the three model datasets. (**g**) Activation curves from the model datasets. (**h**) Example activation maps from unfiltered model datasets with isotropic conduction of 50 cm/s, but with 10% and 30% signal noise. (**i**) Measured CV using multi-vector method (5 × 5 pixel window) from unfiltered (Black), temporally filtered (Savitzky-Golay, Blue), Spatially filtered (3 × 3 Gaussian, Green) and 10 beat ensemble averaged (Red) model datasets at increasing noise levels, n = 5 at each noise level.

**Figure 4:**
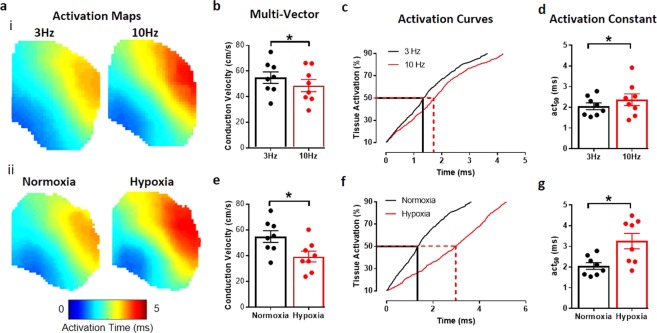
Validation of conduction velocity quantification methods in murine atria. (**a**) Activation maps from murine atria at 3 Hz and 10 Hz pacing (i), and during normoxia and hypoxia (ii). (**b**) Pacing at 10 Hz slows CV, measured using multi-vector method. (**c**) Representative activation curve showing prolongation of activation at 10 Hz vs 3 Hz pacing. (**d**) Pacing at 10 Hz slows CV, measured by time to 50% activation (act_50_). (**e**) CV slowing induced by hypoxia, measured using multi-vector method. (**f**) Representative activation curve showing prolongation of activation curve by hypoxia. (**g**) Hypoxia slows CV, measured by time to 50% activation (act_50_). Grouped data displayed as mean ± standard error, n = 8, *P < 0.05 by paired t-test.

**Figure 5:**
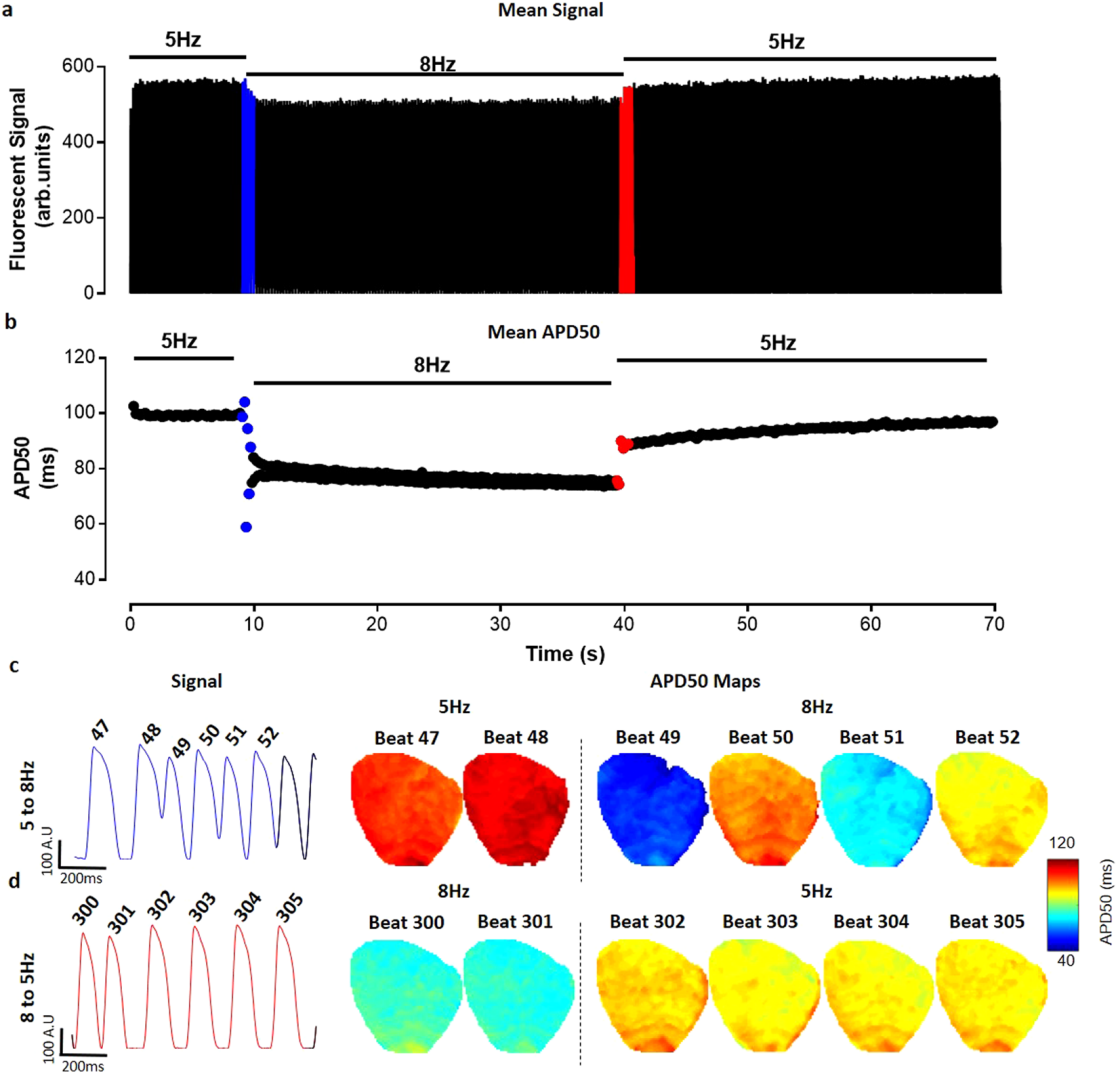
Beat to beat analysis of whole guinea pig heart. (**a**) Tissue averaged fluorescent signal from voltage dye loaded guinea pig heart across 70 s experiment. (**b**) Graph of whole heart beat-to-beat changes in mean APD50. (**c**) Raw trace and whole heart APD50 maps prior to (beats 47 & 48) and immediately after (beats 49–52) pacing frequency increase from 5 Hz to 8 Hz (blue in A and B). (**d**) Raw trace and whole heart APD50 maps prior to (beats 300 & 301) and immediately after (beats 302–305) pacing frequency return to 5 Hz.

**Figure 6:**
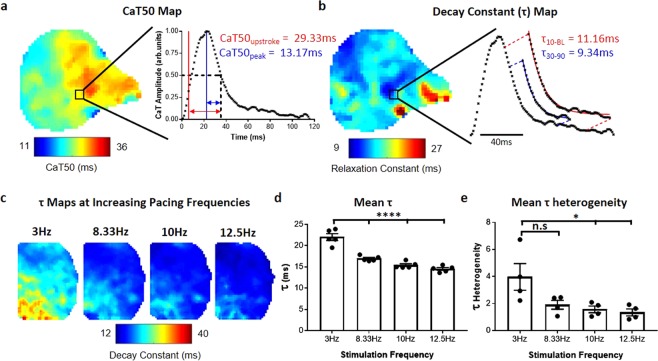
Calcium analysis and decay constant validation. (**a**) CaT50 map from murine atrium as measured from time of maximum upstroke velocity to 50% decay, illustrated in red in region of interest signal (CaT50_upstroke_). Also shown is the measurement of CaT50 from peak amplitude in region of interest (blue, CaT50_peak_ map in Supplementary Fig. VA). (**b**) Map of decay constant, τ, from the same atrium. Map shows calculation of τ by fitting of exponential decay points between 30% and 90% decay from peak (τ_30–90_), illustrated for the regional signal in blue. Also shown is calculation of τ between 10% decay and baseline (red, τ_10-BL_ map in Supplementary Fig. VD). (**c**) Representative τ maps (τ_30–90_) from murine left atria as pacing frequency is increased. (**d**) Data showing shortening of mean τ across the atria with increased pacing frequency. (**e**) Data showing decrease of τ heterogeneity (measured as standard deviation of mean τ across individual atria) across the atria with increased pacing frequency. Data displayed as mean ± standard error, n = 5. P < 0.05, One-Way ANOVA followed by Bonferroni multiple comparison test against 3 Hz pacing.

**Figure 7:**
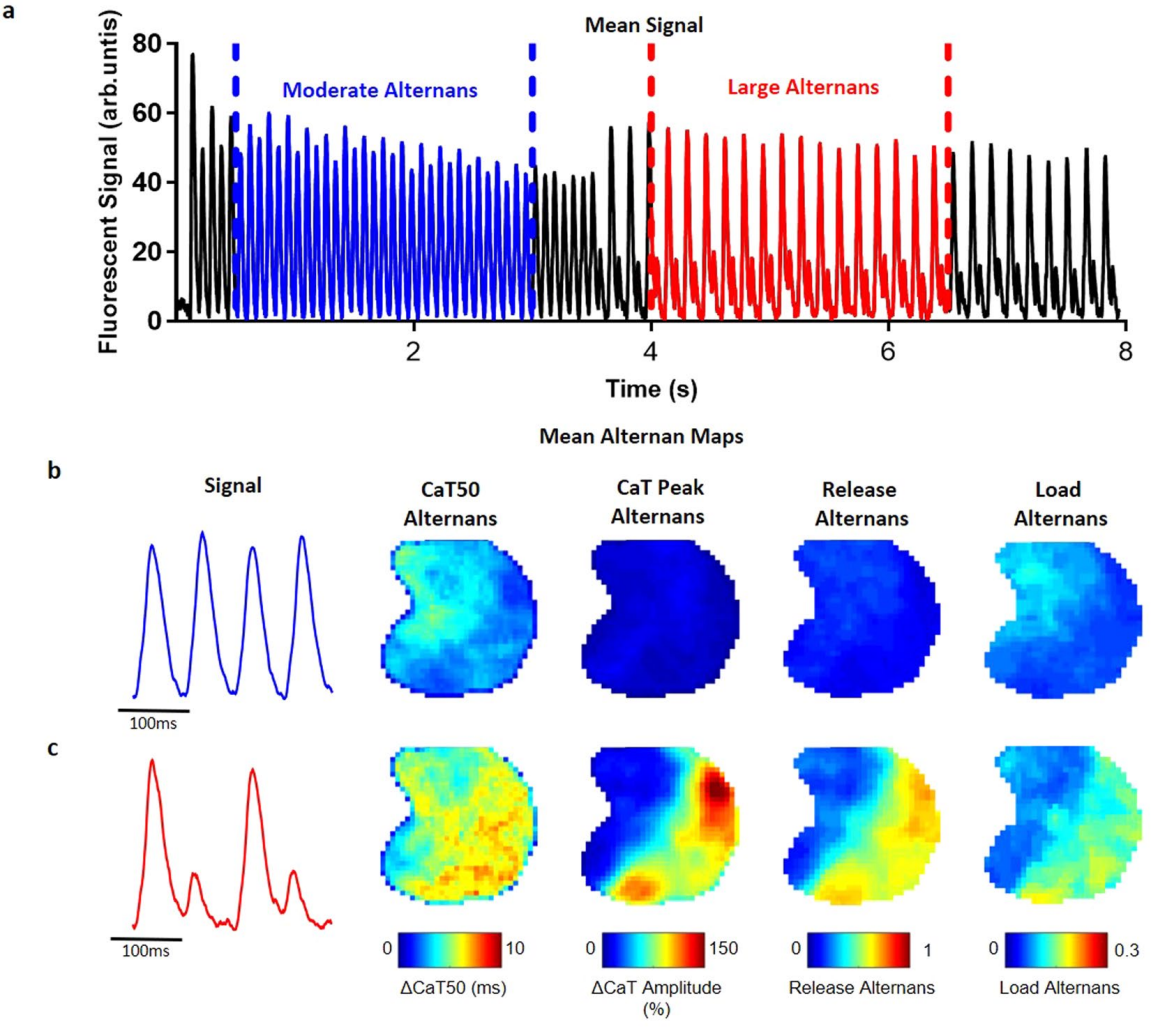
Alternans module. (**a**) Tissue averaged fluorescent signal from calcium dye loaded mouse atrium paced at 12.5 Hz, showing distinct alternans behaviour at 0.5–3 s (blue, moderate alternans), and 4–6.5 s (red, large alternans). (**b**) Representative signals and time averaged alternans maps from tissue during moderate alternans behaviour. Four maps shown correspond to the four measures of alternans available in ElectroMap (see methods and Supplementary Fig. VI). (**c**) Maps during period of large alternans (4–6.5 s) show increase in alternans amplitude, and clear spatial discordance.

**Figure 8:**
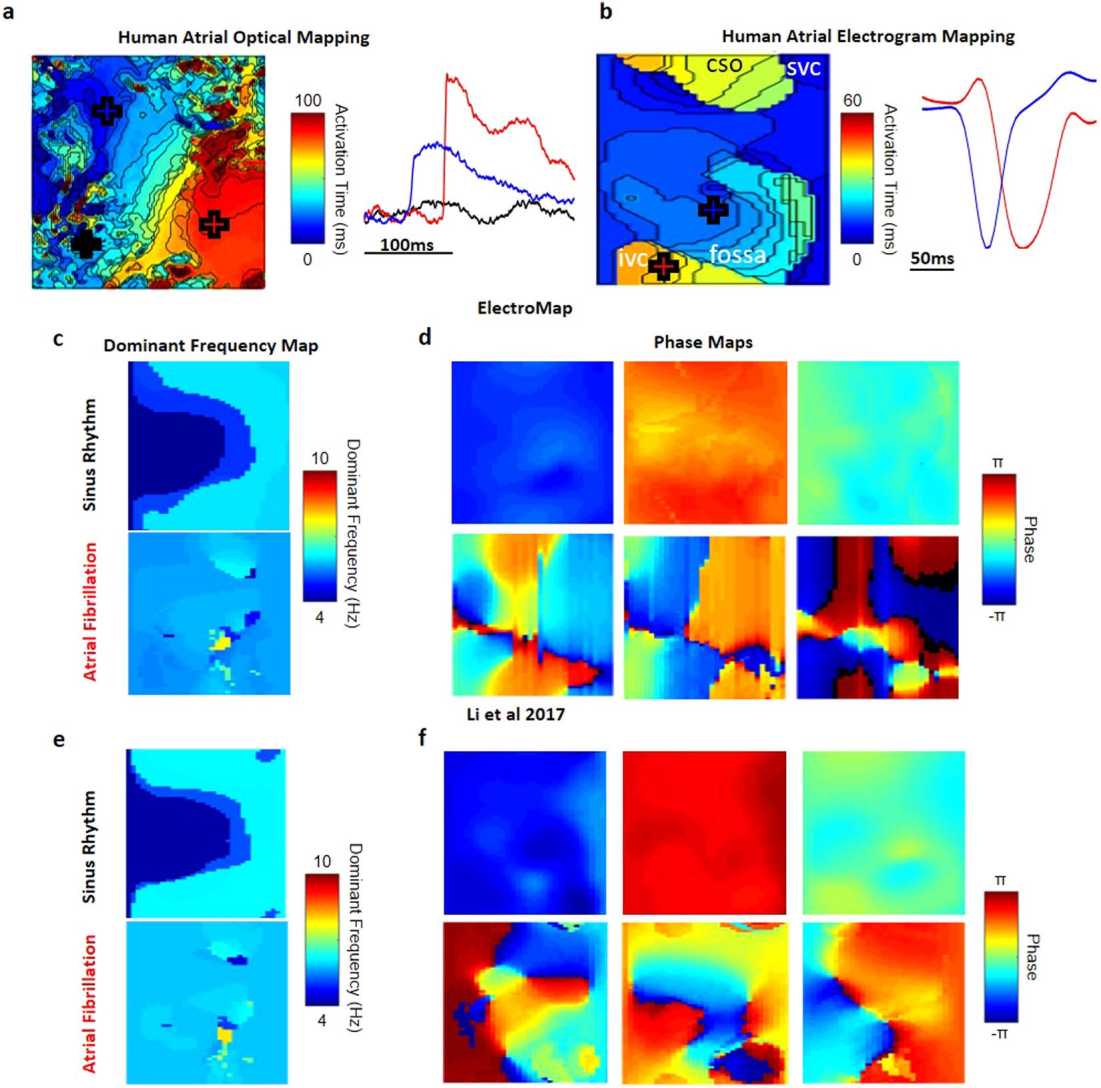
Human atrial optical and electrogram mapping analysis. (**a**) Activation map and example signals generated from human left atrial appendage stained with voltage sensitive dye. (**b**) Activation map and example signals generated from human right atrial *in vivo* electrogram array recordings. Labels refer to closest anatomical structure on 2D map - IVC and SVC: Inferior and Superior Vena Cava, CSO – Coronary Sinus Ostium, Fossa - Fossa Ovalis. (**c**) Dominant Frequency analysis between 4 and 10 Hz, showing localized area of increased frequency during AF. (**d**) Phase maps at 3 time points during sinus rhythm and AF. Synchronised phase behaviour during sinus rhythm is not replicated during AF. The displayed frames are taken from phase mapping over 30 s, Supplementary Video II. (**e**) Dominant Frequency analysis and (**f**) Phase maps at 3 time points during sinus rhythm and AF performed using previously published algorithms of similar datasets^22^.

All data processing and analysis was performed using ElectroMap, Fig. 1a. ElectroMap is developed to run on multiple platforms. It can run either within MATLAB or as a standalone executable (.exe for Windows and.dmg for macOS) with download of the freely available MATLAB runtime (see Supplementary Material). Processing parameters and additional modules are adjustable using custom made graphical user interfaces. Software is designed to handle the widely used TIFF and MAT formats. Custom input of sampling frequency and pixel size makes it compatible with various cameras and acquisition systems. ElectroMap allows automatic or adjusted region selection (Fig. 1b) and multiple filtering options (see Supplementary Material for full details of filtering options). Unless stated otherwise, all the signals in Figs 1–8 underwent spatial (4 × 4 pixel Gaussian, sigma = 1.5) and temporal (3^rd^ order Savitzky-Golay) filtering to allow effective EP parameter quantification from single image pixels (Fig. 1c). The ElectroMap user interface permits signal inversion via a simple checkbox option. User-defined settings can be stored and recalled for future analysis.

Integration of automated pacing frequency recognition and numerous user-defined segmentation options (Supplementary Fig. I) enables simple and intuitive application of powerful analysis strategies including beat-to-beat analysis of long experimental files and multi-beat (ensemble) averaging to improve signal-to-noise ratio (SNR). Code optimization resulted in increased processing speed of up to five-times, compared to our previously published algorithms^15^ (Fig. 1d).

ElectroMap incorporates several definitions for activation (Start – d^2^F/dt^2^_max_, Upstroke – dF/dt_max_, Depolarisation Midpoint, Peak) and repolarisation (Downstroke – dF/dt_min_, Repolarization Percentage, End – d^2^F/dt^2^_max_), Supplementary Fig. II. Repolarisation percentage can be set to any custom value between 0 and 100% repolarisation/decay from peak for both repolarization and duration mapping (Supplementary Fig. III). Unless stated otherwise, depolarization midpoint was used to create activation maps, action potential duration (APD) was defined from upstroke to 50% repolarization and calcium transient duration (CaT) was defined from upstroke to 50% repolarization/decay. For calcium transient analysis, τ was calculated by fitting a mono-exponential model to a user defined region of decay from peak cytosolic calcium. Cardiac alternans were measured as change in duration or peak amplitude from one calcium transient to the previous. Additionally, effects of cytosolic calcium load were investigated by comparing ‘load’ and ‘release’ alternans amplitude, as in Wang *et al*.^23^. Analysis results can be exported from ElectroMap’s interface as TIFF files for individual maps, AVI/GIF files for multi-image analyses such as beat-to-beat (see Supplementary Video I), and comma separated value (.csv) files for parameter distribution and beat-to-beat analysis.

### Statistical analysis

Data are presented as mean ± standard error. Differences between group means were examined using two-tailed, paired Student’s t-test or using One Way Analysis of Variance (ANOVA) with Bonferroni test, and were accepted as significant when P < 0.05.

## Results

We have developed a novel, user-adaptable, semi-automated, robust, open-source software ElectroMap using MATLAB, see Fig. 1a for graphical user interface. The software is freely downloadable from https://github.com/CXO531/ElectroMap, and is available as a standalone executable file or as source code which can be run and edited in MATLAB. Algorithms implemented within ElectroMap have previously been validated against monophasic and transmembrane potentials in murine atria^15^. Here, we present the application and further validation of ElectroMap for the analysis and mapping of basic and complex cardiac electrophysiology from optical mapping datasets (Figs 1–8) and endocardial electrograms (Fig. 8).

### Conduction Velocity Module

Analysis of the spread of electrical activation in cardiac tissue at high spatiotemporal resolution is one of the most powerful applications of optical mapping. We have developed a comprehensive CV analysis module within ElectroMap (Supplementary Fig. IV), to overcome a multitude of complexities and potential user-bias associated with CV calculations^18,24^. The module incorporates two established methods that rely on superimposing a vector field upon the activation map: (i) multi-vector and (ii) single vector method (see Fig. 2a,b). Additionally, we have developed a novel method that quantifies time taken to activate a percentage of the tissue. This approach allows calculation of ‘activation constant’ (see Fig. 2c,d), thereby providing a measure of activation spread across the whole tissue.

To validate the three CV calculation methods, we generated computationally simulated model data (Fig. 3a and Supplementary Fig. V; for algorithms, see Supplementary Material). All three methods accurately measured conduction velocities ranging from 10 to 100 cm/s in isotropic model data (Fig. 3a(i)). As shown in Fig. 3b,c, single vector and multi-vector methods demonstrate a linear increase in CV with faster model conduction speed. Activation curve method accurately detected a change in CV (Fig. 3d), increase in model CV led to a reduction in the time taken to activate 50% (act_50_) of the tissue. We also validated CV analysis methods using models of anisotropic activation (Fig. 3aii) and regional conduction block (Fig. 3aiii). Single vector analysis correctly detected slower CV along the transverse (180^0^/360^0^) compared to longitudinal (0^0^/270^0^) direction in anisotropic waves, and conduction slowing adjacent to the region of conduction block (Fig. 3e). Similarly, multi-vector method successfully identified regions of reduced CV in anisotropic waves and severe conduction slowing around the area of conduction block (Fig. 3f). Activation curve analysis was able to detect the slowing of conduction in the anisotropic wave, as evident by a prolongation of the activation curve, and a delay in activation caused by conduction block (Fig. 3g). Additionally, Gaussian noise with incrementally increasing standard deviation (defined as percentage of action potential amplitude, Supplementary Fig. VII) was introduced to isotropic simulated data with CV of 50 cm/s, Fig. 3h. As expected, increased noise in single beat data decreases the ability to accurately calculate CV, with noise levels above 20% resulting in erroneous CV measurements (Fig. 3i). However, SNR can be improved using temporal and spatial filtering or multi-beat ensemble avenging within ElectroMap. Temporal filtering (Savitzky-Goaly filter) did not improve CV measurement, whereas both spatial filtering (3 × 3 Gaussian filter) and ensemble averaging of 10 beats substantially improved CV measurement accuracy.

Further validation was performed using experimental datasets acquired from murine left atrial tissue. We focused on two physiologically relevant stimuli known to reduce CV: an increase in pacing frequency^25^ and acute hypoxia^26^ (Fig. 4a). Indeed, significant slowing of CV, due to increased pacing frequency, was detected by multi-vector method (Fig. 4b, P = 0.0335) and activation curve method, as shown by an increase in the act_50_ (Fig. 4c and D, P = 0.0119). Both methods also accurately detected a more severe slowing of CV caused by exposure to acute hypoxia (Fig. 4e–g, P < 0.0001 and P = 0.0051 respectively).

### High-Throughput Beat-to-Beat Analysis

Studies of beat-to-beat variation can provide important information about short periods of regional or whole tissue electrical instability, e.g. in response to pacing frequency change, pharmacological agents or disease states. Therefore, we developed and tested algorithms to allow high-throughput analysis of beat-to-beat alterations in long experimental files. Guinea pig hearts were paced over 70 seconds with a 5-8-5 Hz pacing protocol^27^ and beat-to-beat changes in APD50 examined (Fig. 5a and Supplementary Videos I and II). Increasing the pacing frequency (from 5 Hz to 8 Hz) induced reversible shortening of mean ventricular APD (Fig. 5b). Furthermore, significant APD heterogeneity was detected in the first 4 beats, immediately after transition from 5 Hz to 8 Hz (Fig. 5b,c). Switching back from 8 Hz to 5 Hz (at 40 s) also induced some APD heterogeneity while highlighting a slow return to the steady state APD (Fig. 5b,d).

### Calcium Decay Mapping

Optical mapping is increasingly utilised to study calcium release patterns across myocardial tissue or cellular monolayers, in response to disease, genetic factors or drug administration^19^. The estimation of calcium decay constant (τ) is common in single cell studies. However, τ is not commonly mapped across multicellular preparations^28^, likely due to lack of support for this function in available software platforms. We developed algorithms for calculation of CaT and τ maps, as shown in Fig. 6a,b. Importantly, CaT can be measured from various ‘activation times’ during upstroke (Supplementary Figs II and VI), allowing for assessment of both calcium release and uptake (Fig. 6a inset). Moreover, τ can be fitted from any user-defined value (Fig. 6b inset). To test and validate algorithms developed for calculation of τ, mouse atria were loaded with Rhod-2AM and paced at incremental frequencies (3-8.33-10-12.5 Hz). Software accurately calculated progressively shorter τ values at higher pacing frequencies (Fig. 6c,d, P < 0.0001 for all pacing frequencies compared to 3 Hz). Interestingly, higher pacing frequencies were associated with a reduction in τ heterogeneity (Fig. 6e, P = 0.0286 and P = 0.0168 for 10 Hz and 12.5 Hz pacing respectively).

### Alternans Analysis Module

Alternans are beat-to-beat 2-period oscillations in ion handling, electrical activity and hence mechanical contraction of the myocardium that can act as a precursor to conduction block and lethal arrhythmias^29,30^. We have developed a module within ElectroMap to detect and quantify alternans across tissue and cellular monolayers. Alternans module is capable of quantifying peak, duration, and release or load alternans (see Supplementary Fig. VIII for alternans definitions).

In Fig. 7 we demonstrate utility of the alternans module in detection and quantification of calcium alternans in a murine left atrium loaded with Rhod-2AM. The atrium was subjected to high frequency pacing of 12.5 Hz, initially leading to moderate alternans (Fig. 7a, blue CaTs) and then transitioning to more pronounced alternans (Fig. 7a, red CaTs). ElectroMap was used to generate maps allowing visualization and alternans quantification across the atrium (Fig. 7b,c). Alternans displayed clear spatial heterogeneity (Fig. 7b,c) with increased ‘CaT Peak Alternans’ correlating with changes in diastolic calcium load (‘Load Alternans’), see Fig. 7c.

### Human Optical Mapping and Electrogram Recordings

ElectroMap’s utility extends to human tissue, see Fig. 8a for optical mapping of human atrial appendages. We also looked to test whether ElectroMap can be applied to analysis of clinically relevant mapping technologies. Right atrial virtual electrograms from a patient during sinus rhythm and AF were recorded and analysed after QRST subtraction^22^. During sinus rhythm, the spread of activation was calculated from the timing of dV/dt_min_ in each electrogram, generating an activation map (Fig. 8b). ElectroMap clearly identified higher DF components (Fig. 8c) during AF in the physiological range of 4–10 Hz (DF range can be set within ElectroMap’s interface). Additionally, marked phase discontinuities (Fig. 8d and Supplementary Video III) are observed in the electrogram recordings during AF. These findings are confirmed using previously published algorithms^22^ with similar areas of high dominant frequency and discontinuous phase behaviour identified (Fig. 8e,f).

### Additional Features

ElectroMap analysis options further extend to measure diastolic interval (DI) and time to peak. DI is measured from APD90 to the following activation time, which can be difficult to quantify in low SNR samples on a single beat. To validate the DI function, guinea pig hearts were loaded with Di-8-ANEPPS and pacing cycle length was decreased in 10 ms intervals from 170 ms to 110 ms. Ensemble averaging of 10 beat segments was used to produce an ‘average beat diastolic interval’, with improved SNR, and DI mapping function was validated by plotting DI-APD restitution curves (Supplementary Fig. VIII). Both APD50 and DI decrease more rapidly at higher pacing frequencies as previously demonstrated^31^.

Optogenetic excitation and pacing via light pulses in cardiac tissue expressing light sensitive ion channels is a novel research tool^32,33^ and may even be used for cardiac pacing in patients^34^. However, introduction of large amplitude light pulses will distort optical mapping images and thus preclude processing and analysis of such data. To this end, ElectroMap integrates a pacing artefact removal algorithm (Supplementary Fig. IX), which can identify and correct for light-pacing peaks, thereby allowing processing and analysis of previously obscured data.

## Discussion

This work presents the development and validation of a novel open-source software for analysing and mapping optical and electrical signals. We demonstrate the compatibility of ElectroMap with a variety of camera types, species and experimental models. Key features include comprehensive measurement, analysis and mapping of global and regional conduction, versatile signal segmentation and semi-automated high-throughput analysis of action potential and its spatial and temporal (beat-to-beat) variations, calcium transients, and cardiac alternans. Using model and real data, we demonstrate the application of ElectroMap for analysis, measurement and mapping of basic and complex electrophysiology and its utility in dissecting pro-arrhythmic mechanisms *in vitro* and *in vivo*.

ElectroMap is built on years of international research and development in optical mapping processing and analysis, described in many excellent papers and reviews^6,14,15,18,35,36^. Algorithms that ElectroMap relies on have previously been validated against monophasic and transmembrane potentials in murine atria^15^. Further validation performed against established open-source (Rhythm^14^) and commercially available software (Optiq, Cairn Research, UK) yielded similar APD values and activation maps (Supplementary Fig. X). To the best of our knowledge, this is the first such comparison of commonly used analysis algorithms, which should help instil confidence in interpretation of data generated thus far. Importantly, ElectroMap provides further functionalities and outputs, not widely available currently, including ensemble averaging, multiple CV methodologies, automatic frequency detection, signal segmentation, alternans analysis and decay constant mapping. The use of ElectroMap extends beyond optical mapping, allowing for its application in clinical settings with electrogram array data.

Robust validation studies performed using both model and experimental data demonstrate ElectroMap’s utility for accurately quantifying key parameters of electrical function and calcium release across cardiac tissue. CV quantification in model data with noise verified that processing using ElectroMap allows accurate parameter quantification. In this case, ensemble averaging proved the most effective processing strategy due to the randomness of the noise and the underlying identical morphology of all simulated action potentials. In other circumstances, (e.g. beat-to-beat variations) ensemble averaging of signals should be avoided, necessitating distinct processing strategies such as spatial filtering, temporal filtering and baseline correction which are all employable using ElectroMap.

Additionally, we demonstrate that improved processing speeds with automatic or user defined signal segmentation enable more complex and computationally challenging analyses; e.g. beat-to-beat variations in APD or regional differences in calcium decay. Indeed, we demonstrate that using ElectroMap, rapid and straightforward analysis of long experimental datasets can reveal acute periods of pro-arrhythmic EP behaviour throughout the entire guinea pig myocardium (Fig. 5). Such analysis would otherwise be missed by ensemble averaging of multiple beats or analysis of a single beat. ElectroMap, in contrast, provides rapid analysis of these complex EP parameters and exports to video and spreadsheet formats that facilitate interpretation of the observed patterns. This may be of particular importance when examining the pathophysiological effects of rapid changes in heart rate and/or immediate and prolonged responses to stimuli such as adrenaline or acute ischaemia. With improving SNR through better cameras and dyes, there is an increasing demand for beat-to-beat analysis of mapping data^37,38^. We anticipate that ElectroMap’s ability to achieve such analysis in a high-throughput semi-automated manner will prove valuable for preclinical and clinical researchers.

The ability to simultaneously use three CV quantification methods is a key feature of ElectroMap. This is essential due to inherent limitations of each individual methodology for analysis of cardiac CV^18,24^. Here, we demonstrate that single vector method can suffer from significant user-generated overestimation of CV (see Supplementary Fig. IVA). More automated approaches have been proposed, such as that of Doshi *et al*.^39^ whereby single vector quantification is limited to areas where activation time linearly increases. Within ElectroMap’s conduction module, we have looked to overcome and highlight difficulties with the single vector method via an automatic angular sweep of measured CV from a user chosen point, as in Fig. 3e and Supplementary Fig. IVA. This approach reveals inherent variability that stems from user-defined propagation direction. ElectroMap automatically identifies the slowest conduction direction, which, in normal circumstances, will be parallel to wavefront propagation. The angular sweep additionally allows straightforward identification of longitudinal and transverse CVs^40,41^.

Application of multi-vector method reduces the likelihood of user-introduced errors but does not eliminate them completely. Simulation studies by Linnenbank *et al*.^18^ for instance demonstrate that despite increased automation, multi-vector methods can introduce systematic errors in CV quantification. The successful use of the method requires minimum criteria to be met in terms of grid size, local region size and angular binning for identification of propagation directions. Another problem can stem from inability to fit local vectors to some areas of the tissue. For example, activation definitions that are intrinsically limited to sampling rate (such as upstroke) can result in local regions where measured activation time is the same throughout. In such circumstances, meaningful surface fit (e.g. Fig. 2b) or other methods for describing local activation such as finite difference analysis^42^, are not achievable^24^. This can be particularly problematic in small tissue samples, like isolated murine left atria, or in tissues exhibiting fast conduction and/or wavebreak. Viable solutions to this issue include interpolation of data between sampling points or use of alternative activation definitions such as depolarisation midpoint (see Supplementary Fig. II and IV). However, this relies on accurate signal interpolation, not always achievable in practice^43^. Epicardial conduction velocity quantification can also be complicated by multiple breakthroughs in the endocardium leading to inaccurate CV estimation during complex arrhythmias^44^ or in the absence of epicardial pacing. In conclusion, there is no universally applicable activation measure suited for every optical mapping experiment, further validating the need for integration of multiple activation and CV quantification methodologies within ElectroMap.

ElectroMap incorporates a novel activation curve method of conduction analysis. This method allows users to quantify the time taken to activate a defined tissue percentage or study the whole activation curve. The methodology is less susceptible to user bias or to sparsity of local vectors in areas of rapid conduction as it does not rely on user input or vector calculation. Furthermore, activation time can be normalized to tissue size, yielding an activation constant in units such as ms/mm^2^. This extends the use of the activation constant to studies where tissue morphology may be altered, such as in hypertrophy or genotype dependent differences in cardiac structure. However, activation constant only provides a summarised measure of activation, thus a combination of analysis methods may be required for robust interrogation of cardiac conduction. Additionally, despite the various CV methods within ElectroMap, conduction analysis can remain challenging if the underlying activation pattern is heterogeneous, often observed in arrhythmic tissue and cultured cardiac monolayers^45^. The ‘Ccoffinn’ method of Tomek *et al*.^24^ combines sophisticated image processing techniques with novel wavefront tracking algorithms and can potentially overcome heterogeneity issues^24^.

Defining activation times for APD and CaT duration mapping is an important but complex and often poorly understood analysis option. Different studies utilize distinct features of the upstroke to define activation, including dF/dt_max_ (upstroke)^44^, d^2^F/dt^2^_max_ (start)^46^, depolarization midpoint^47^ and peak^8^. Similarly, time of repolarisation/decay can be measured at a defined repolarisation percentage^15,48^, dF/dt_min_ (downstroke) or d^2^F/dt^2^_max_^36^. The parameters best suited for duration measurement will depend on experimental model, signal quality and experimental question. We have integrated all of these user-selectable definitions within ElectroMap for both APD (Supplementary Fig. II) and CaT mapping (Fig. 6a and Supplementary Fig. VI).

Similarly, τ calculations can be customised in ElectroMap’s interface to calculate decay constant for any user-defined segment of the calcium reuptake. This permits analysis of cytosolic or SR^23^ calcium handling kinetics in different animal species and cells. For example, note the regional discrepancy between CaT50 and τ atrial maps in Fig. 6a,b. This contrast results from the definition in CaT50 used, i.e. time from upstroke to 50% decay (CaT50_upstroke_). CaT50_upstroke_ is a measure of both the release and reuptake of the cytosolic calcium. In contrast, τ depends only on reuptake kinetics, and so is unaffected by the changes in calcium release. By measuring CaT from peak (CaT50_peak_), the CaT50 and τ values become more similar across the atria (Supplementary Fig. VI). Deciphering pathophysiological relevance of these observations is not within the remit of this manuscript. However, we predict the user-defined tools for comprehensive measurement of vital duration and relaxation parameters that ElectroMap provides will aid understanding of cardiac pathophysiology.

The utility of processing and analysis algorithms in ElectroMap can extend beyond optical mapping. Here, we show analysis of *in vivo* virtual unipolar electrogram recordings from human right atrium (Fig. 8b–d). Despite the contrasting waveforms compared to optical mapping, by analysing the timing of dV/dt_min_ in electrogram recordings, ElectroMap is able to accurately calculate local activation times. Beyond its clinical utility, access to analyses such as dominant frequency and phase mapping, allowing mapping the site of triggered activity during arrhythmia (Fig. 8c,d), can aid the translation of experimental data^10,49^. These capabilities of ElectroMap require further systematic validation against a range of clinically used platforms, however current outputs are comparable to previous published analysis of similar datasets^22^, see Fig. 8c–f. Implementation of other analysis options available within ElectroMap such as beat-to-beat segmentation, CV quantification and alternans detection and quantification hold immense potential for the analysis of electrogram data.

ElectroMap provides a robustly validated open-source flexible tool for processing and analysis of optical mapping data. We anticipate ElectroMap to facilitate increased uptake of optical mapping in cardiac electrophysiology. Furthermore, application of novel data analysis strategies developed here will further our understanding of the mechanisms underpinning lethal arrhythmia. The application of ElectroMap extends beyond optical mapping. Here, we have demonstrated that ElectroMap can also be used in the analysis of clinical electrogram-based mapping data. Moreover, application of high-throughput CV quantification methodologies can be applied to drug-screening pipelines for cardiotoxic effects in cell monolayers. There is scope to broaden the existing functions of ElectroMap, for example, by introducing dual-analysis of simultaneous voltage and calcium recordings and implementing computational approaches to overcome motion artefacts^50^. Furthermore, increases in computational efficiency through more sophisticated computational techniques, such as parallel processing, would allow for faster data processing and could eventually facilitate in-acquisition analysis.

## Supplementary information

Video I

Video II

Video III

Supplementary Information
