# Supplementary material for: ElectroMap: High-throughput open-source software for analysis and mapping of cardiac electrophysiology
Source: Sci Rep. 2019 Feb 4;9:1389. doi: 10.1038/s41598-018-38263-2 (PMC6362081; doi:10.1038/s41598-018-38263-2)
Supplement: Supplementary file 4 — Supplementary Information [file 41598_2018_38263_MOESM4_ESM.pdf]

SUPPLEMENTAL MATERIAL

ElectroMap: High-throughput open-source software for analysis and mapping of cardiac electrophysiology

Christopher O'Shea, Andrew P Holmes, Ting Y Yu, James Winter, Simon P Wells, Joao Correia, Bastiaan J Boukens, Joris R de Groot, Gavin S Chu, Xin Li, G.Andre Ng, Paulus Kirchhof, Larissa Fabritz, *Kashif Rajpoot, *Davor Pavlovic

Detailed Methods

Software Development and Availability

All software, processing algorithms and model datasets were developed and implemented in MATLAB R2017a (Mathworks, USA), see Figure 1A for graphical user interface (GUI). GUI was designed using MATLAB's GUIDE functionality, and the software can be run either within MATLAB (assuming required toolboxes are present) or from a standalone executable file (e.g. .exe on a Windows PC, .dmg for Mac OS). Use of the executable file will require download of the freely available MATLAB Runtime (https://uk.mathworks.com/products/compiler/matlab-runtime.html). Total download size ~ 1.3GB. To run the software within MATLAB, the Image Processing, Signal Processing, Statistics and Machine Learning and the Curve Fitting toolbox are required. The total download size of MATLAB code (.m) files < 3MB.

Software source code can be retrieved at https://github.com/CXO531/ElectroMap while executable versions are hosted at https://drive.google.com/open?id=1nJyI07w9WIt5zWcit0aEyIbtg31tANxI. The software was developed and optimised for use on windows machines and this is the recommended operating system for the software. However, the software has also been tested and successfully used on MAC machines.

Optical Mapping

Mouse (MF1 n=8, 129/Sv n=1) optical mapping experiments were conducted as previously reported[1–3]. Animals were anaesthetised using inhaled 4% isoflurane in O_2. Whole hearts were then isolated, mounted on Langendorff apparatus and perfused via the ascending aorta. A standard bicarbonate buffered Krebs–Henseleit solution at 37°C was used containing in mM: NaCl 118; $NaHCO_3$ 24.88; KH_2PO_4 1.18; Glucose 5.55; Na-Pyruvate 5; $MgSO_4$ 0.83; $CaCl_2$ 1.8; KCl 3.5. Fluorescent dye in DMSO (Voltage: DI-4-ANEPPS (0.125mg/ml), Calcium: Rhod-2-AM (1mg/ml)) was then loaded for 5-10 minutes. The left atrium was then isolated, and the posterior atrial surface exposed. The atria were superfused under normoxic (95%O_2/5%CO_2) or hypoxic (95%N_2/5%CO_2) conditions with Krebs solution containing contraction uncoupler blebbistatin (10µM) and paced with bipolar platinum electrodes at 2 times diastolic threshold. Fluorescent dyes were excited by four LEDs at 530nm, and filtered fluorescence (>630nm for DI-4-ANEPPS; 565-605nm for Rhod-2-AM) captured using ORCA flash 4.0 camera (1kHz sampling rate, 200x2048 pixels, 71µm/pixel).

Optical mapping experiments in guinea pigs (n=6) were conducted as previously reported[4,5]. Adult male guinea pigs were anaesthetised with sodium pentobarbitone (160mg/Kg, i.p.) along with concomitant heparin. Hearts were perfused in Langendorff mode through the descending aorta (perfusion pressure 65-75mmHg, 37°C). Buffer solutions contained in mM: NaCl 114, KCl 4, CaCl 1.6, $NaHCO_3$ 24, $MgSO_4$ 1, NaH_2PO_4 1.1, glucose 11.0, sodium pyruvate 1.0 and decamethonium bromide 0.01. Solutions were filtered using an in-line cellulose filter (5µm pore diameter)). Hearts were uncoupled with blebbistatin (15µM) and stained with Di-8-ANEPPS (1mg/ml in DMSO; 200-300µL). Dyes were injected slowly into the perfusion line over a 5 to 10-minute period. Excitation light was at 530nm, with emission light collected at >610nm. Hearts were imaged through an Olympus MVX10 stereomicroscope and signals recorded on Evolve Delta 512x512 pixel EMCCD cameras (500Hz sampling rate, 64x64 pixels, 320µm/pixel).

Optical mapping experiments of human atria were conducted as previously reported[6]. The left atrial appendage was removed using an endoscopic stapling device (Endo Gia stapler, Tyco Healthcare Group). The tissue sample was transported to the optical mapping setup in 100-mL cooled superfusion fluid (Na^+, 155.5 mmol/L; K^+, 4.7 mmol/L; Ca^{2+}, 1.45 mmol/L; Mg^{2+}, 0.6 mmol/L; Cl^-, 136.6 mmol/L; HCO_3^-, 27 mmol/L; PO_4^{3-}, 0.4 mmol/L; glucose, 11.1 mmol/L; and heparin, 1000 IE. In the optical mapping setup the preparation was submerged in a tissue bath. The superfusion fluid was kept at a temperature of 36.5°C to 37.5°C and oxygenized with a mixture of 95% O_2 and 5% CO_2 to maintain a pH of 7.4. The preparations was stimulated at 100 beats per minute at twice diastolic threshold with a pulse width of 2 ms using an epicardial electrode. The preparation was equilibrated for ≥30 minutes. Di-4-ANEPPS (Tebu Bio) was used as a membrane potential-sensitive fluorescent dye and Blebbistatin to remove motion artifacts. A MiCAM Ultima camera (SciMedia USA Ltd) was used to record epicardial images of an area of 1 cm^2 with a resolution of 100×100 pixels and a sample time of 0.5 ms. Images were stored using MiCAM Ultima Experiment Manager. The occurrence of small motion artifacts precluded analysis of the repolarisation across the atrial preparation.

Processing and Analysis Parameters

The software allows a choice of spatial filtering, temporal filtering, and baseline correction methods. However, in the analysed data presented in this work, a 4x4 pixel Gaussian spatial filter was applied to the raw signal which was subsequently filtered using a 3rd order Savitzky-Golay temporal filter. Linear top-hat filtering was used to correct for non-physiological changes in baseline fluorescence.

Results were exported from ElectroMap's interface in a variety of formats depending on specific analysis performed. To study both mean values but additionally tissue heterogeneity for conduction velocity and calcium decay constant, all calculated values within the maps were exported as comma separated value files, as were the results of angular distributions of CV and the activation curves. The corresponding maps were exported as TIFF files. For extended beat-to-beat analyses, summary data (mean, standard deviation) for each individual beat was exported in a single comma separated value as it was calculated automatically in ElectroMap once the whole file analysis had begun. Corresponding maps at each individual beat were also exported as a single GIF file and additionally AVI video files.

Activation and duration mapping

For the quantification of conduction velocity, it is necessary first to define time of activation for each pixel in an image to create the activation map. Equally, activation time requires measurement to quantify both action potential and calcium transient duration. For the methods for this are employable in ElectroMap, see Supplementary Figure I. For conduction velocity analysis, unless otherwise stated, the midpoint of depolarization was used to produce the activation maps. APD and CaT measurements were taken from upstroke (i.e. dF/dt_{max}) to 50% repolarisation/decay, unless otherwise stated.

Conduction Velocity measurement

A stand-alone conduction velocity module was developed and integrated within ElectroMap, including two established methodologies for CV calculation, 'multi-vector' and 'single vector' methods (figure 3A and B) while a novel activation curve method (figure 3C and D) is introduced.

The polynomial 'multi-vector' method, modified for use from epicardial mapping data, spatially segments the activation map into regions of n x n pixels[7]. For each of these local regions, a polynomial surface, $f(x,y)$, is fitted that best describes the relationship between activation time, t, and spatial position (x,y). The gradient vector, CV_{local} of this surface can then be calculated as:

$$CV_{local} = \nabla f(x,y) \qquad (1),$$

where ∇ denotes the two-dimensional cartesian spatial differential operator. This vector points in the direction of propagation defined by the surface, and so describes both the local magnitude and direction

of conduction[7]. Mean conduction velocity can then be calculated as the average magnitude of the local vectors.

The 'single vector' method will measure the time delay (Δt) in activation between two points, and then use the distance between the points (r) to estimate conduction velocity. The single vector velocity, CV_{single}, between two points separated by distance r will then be calculated as:

$$CV_{single} = r/\Delta t \quad (2).$$

Additionally, we have developed and implemented a novel 'activation-curve' method for conduction analysis. Here, the percentage of tissue activation is plotted as a function of time as demonstrated in Figure 2D. Hence, at time t, the value of the activation curve as a percentage, $Act(t)$ will be:

$$Act(t) = 100 * \frac{1}{N} . \sum_{n=1}^{N} \delta_n(t), \quad \delta_n(t) = \begin{cases} 1, if\ pixel\ n\ activated\ by\ time\ t \\ 0, otherwise \end{cases} \quad (3)$$

in a tissue image series made up of N pixels. Equation 3 can similarly be used to plot a repolarisation curve, where $\delta_n(t)$ is redefined to become 1 at time of repolarisation. From this activation curve, it is possible to define an activation constant, such as time to reach 50% (act_{50}) or 90% (act_{90}) activation. Comparison between activation constants of each tissue can then allow objective comparison of changes in conduction kinetics.

<u>Calcium decay constant (τ)</u>

τ was calculated by fitting of a mono-exponential decay model of the form:

$$F(t) = F_0 e^{-t/\tau} + C \quad (4)$$

where the fluorescence level at time t depends on the peak fluorescence, F_0, and the subsequent decay (C is a constant). For the calculation of τ present in Figure 6, equation 4 was fitted from 30% to 90% ($\tau_{30\text{-}90}$) decay from peak cytosolic Ca^{2+}. However, we also investigated the effects on decay constant mapping of fitting to other stages of Ca^{2+} decay, such as from 10% to baseline ($\tau_{10\text{-}BL}$, Supplementary Figure VE).

<u>Cardiac alternans module</u>

Cardiac alternans were defined by four different measures, duration alternans, peak alternans, load alternans and release alternans (Supplementary Figure VI). Peak alternans (ALT_{peak}) between subsequent calcium transients was defined as the percentage increase in peak value of the larger peak ($peak_l$) compared to the smaller peak ($peak_s$):

$$ALT_{peak} = 100 * \left(\frac{peak_l}{peak_s}\right) \quad (5)$$

Duration alternans (ΔAPD or ΔCaT) were measured as the time difference between the duration of one action potential/calcium transient and the next. For example, in the case of two consequent calcium transients of duration CaT_1 and CaT_2 :

$$\Delta CaT = |CaT_1 - CaT_2| \quad (6).$$

The effects of cytosolic Ca^{2+} load and overall amplitude (in contrast to just peak value) were investigated by comparing the load alternans amplitude to the release alternans amplitude, as in Wang et al., 2014 (where similar measures were applied to study sarcoplasmic reticulum Ca^{2+} handling)[8]. If L is defined as the peak amplitude of the large beats (i.e. where the amplitude is greater than the previous beat), S the amplitude of the small beats, and D the diastolic load of the small beats the release alternans ($ALT_{release}$) are defined as:

$$ALT_{release} = 1 - (S/L) \quad (7).$$

Conversely, we define load alternans (ALT_{load}) as:

$$ALT_{load} = D/L \quad (8).$$

Dominant frequency and phase mapping

Dominant frequency analysis is achieved by calculating the frequency spectrum of the input signal by fast Fourier transform. Dominant frequency was then defined as the frequency with the most power in the resulting frequency spectrum in the range of 4-10Hz. Zero padding was applied to deliver a power spectrum with a frequency resolution of 0.05Hz[9,10]. Settings such as frequency range and resolution are controlled within ElectroMap's interface. Phase mapping is performed by calculating the Hilbert transform of the signal, from which the instantaneous phase ranging from -π to π can be calculated at a given time point[10,11].

Production of model murine datasets

The datasets were produced by creating a MATLAB function that for a given time, t, will return the fluorescence value $F(t)$. This function acts by simply approximating the murine optical action potential (i.e. that recorded by a potentiometric dye) in a piecewise manner. Depolarization of the action potential was modelled using a simplified Gaussian function:

$$F(t) = F_0 e^{-(t-t_p)^2} \; ; \; 0 \le t \le t_p \quad (9)$$

where t_p is the time to peak, i.e. time between depolarisation starting and peak transmembrane voltage and F_0 is the maximum fluorescence/voltage value. The repolarisation was then modelled in two parts:

$$F(t) = m * (t - t_p) + F_0 \; ; \quad t_p < t \le (t_p + apd20) \quad (10)$$

where m is the line gradient that connects the peak fluorescence at t_p to a later time $(t_p + apd20)$ where the fluorescence is 80% of its peak value (as dictated by the user, $apd20$). Hence the early repolarisation is modelled as a straight line. From this point, it is then modelled as an exponential decay:

$$F(t) = Ae^{Bt}; \; t > t_p + apd20 \quad (11)$$

where A and B are constants found by fitting an exponential decay to user defined values for the times of 30%,50%,70% and 90% repolarisation occur (i.e. apd30, apd50, apd70, apd90). Random noise was added to $F(t)$ by selecting a value from a set of normally distributed pseudorandom numbers, the standard deviation of which is set as a percentage value of the action potential amplitude. An example of using this model function is shown in Supplementary Figure IVA, as well as the effect of different noise levels on simulated action potential morphology (Supplementary Figure IVB).

2D images of a murine atria have then been modelled using this function. The pseudocode for this is shown below, where factors such as overall velocity, the presence of a conduction block and sampling rate are used to work out an 'effective time' for each pixel in each frame. This model has several limitations owing to its simplicity both in approximation of the murine action potential (a morphological approximation) and conduction across the atria. Therefore, it is currently limited to the simple validations presented here.

Model Pseudocode

```
For each frame
Compute frame time, T = Frame_number*(1/sampling_rate)
    For each pixel
        If pixel not within area of conduction block
            Find effective distance,
            D = (x^2 + (A*y)^2)^(1/2)
            where x and y are coordinate distance from wave origin (centre of image) and
            A is a constant that controls the degree of anisotropy in conduction. For
            example, A=1 represents isotopic conduction, A=2 represents 2 times slower
            conduction along y direction compared to x.
        End
        If pixel inside area of conduction block
            Find effective distance
            D=D1+D2.
            where D1 = effective distance before reaching conduction block and D2 =
            effective distance within conduction block. Hence
            D1=(x1^2 + (A*y1)^2)^(1/2)
            where x1 and y1 are coordinate distance from origin to area of conduction and
            D2=C* (x2^2 + (A*y2)^2)^(1/2)
            where x2 and y2 are coordinate distance from edge of conduction block region
            along line connecting pixel with origin. C is a constant that represents the
            conduction block factor, i.e. C=2 represents 50% velocity within block region
            compared to rest of tissue.
        End
    Compute effective time, t=T-D/V
    where V is the velocity of wave propagation.
    Compute F(t) using function described above and save F(t) for each pixel within each frame.
    Addition of noise
End
```

Supplemental Figures

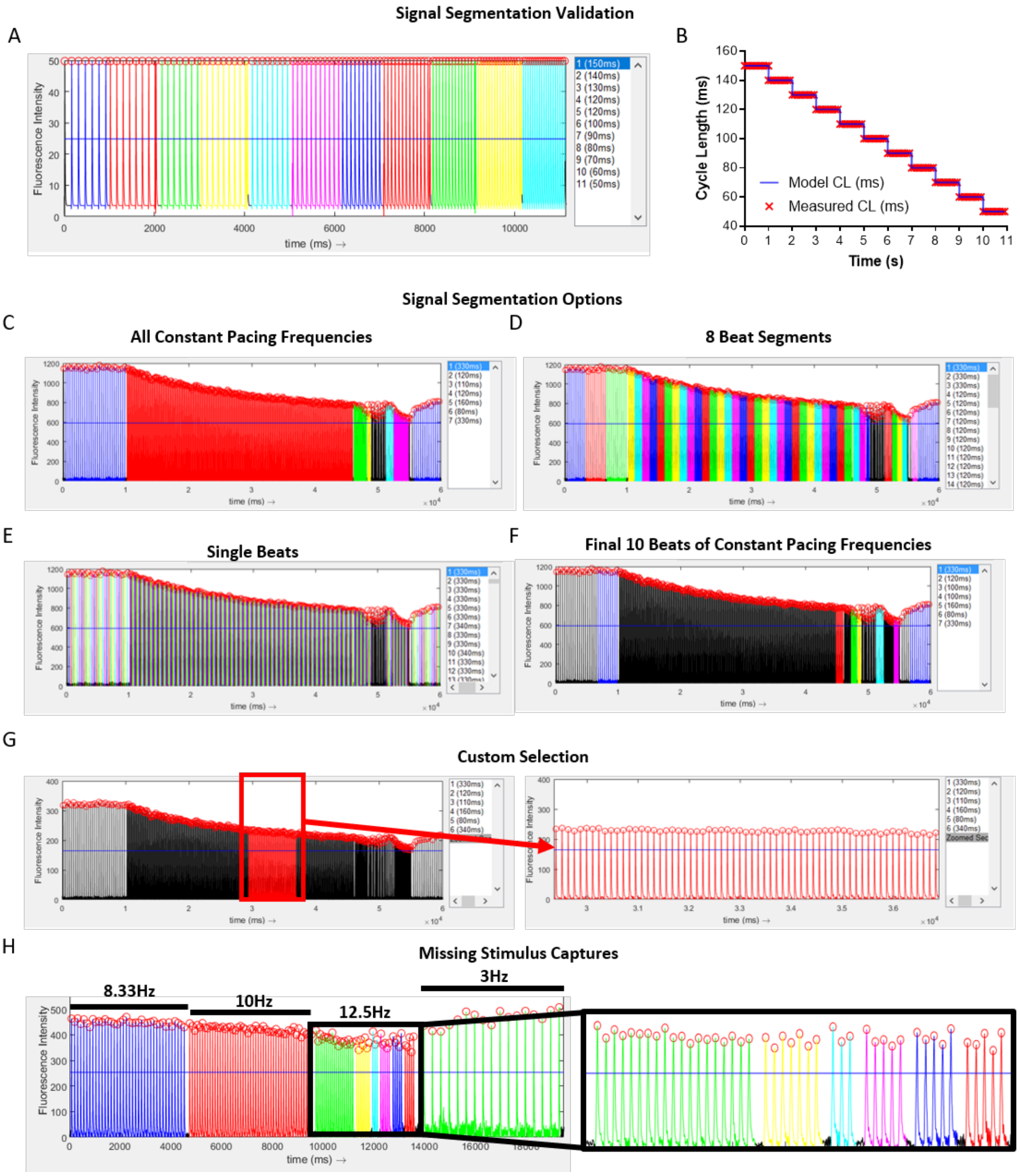

Supplementary Figure I. Automatic, pacing frequency, based signal segmentation options and validation. A & B) We validated our pacing frequency-based segmentation by created a model dataset where the cycle length was reduced by 10ms every 1s from 150ms to 50ms (A). B) The measured CL in ElectroMap exactly matched that of the model throughout. C-G) ElectroMap automatically detects changes in pacing frequency in real experimental data, as demonstrated in (C). The signal can then be further segmented into segments of a desired number of beats, such as 8 shown here in (D). This can be done down to a single beat level (E). Equally, only the final few beats at a given pacing frequency can be analysed to allow sufficient frequency adaption, such as the final beats in (F). G) Along with automatic segmentation, custom region selection is achievable by selecting the desired time from the signal. H) Example of automatic segmentation with missing stimulus captures induced by 12.5Hz pacing.

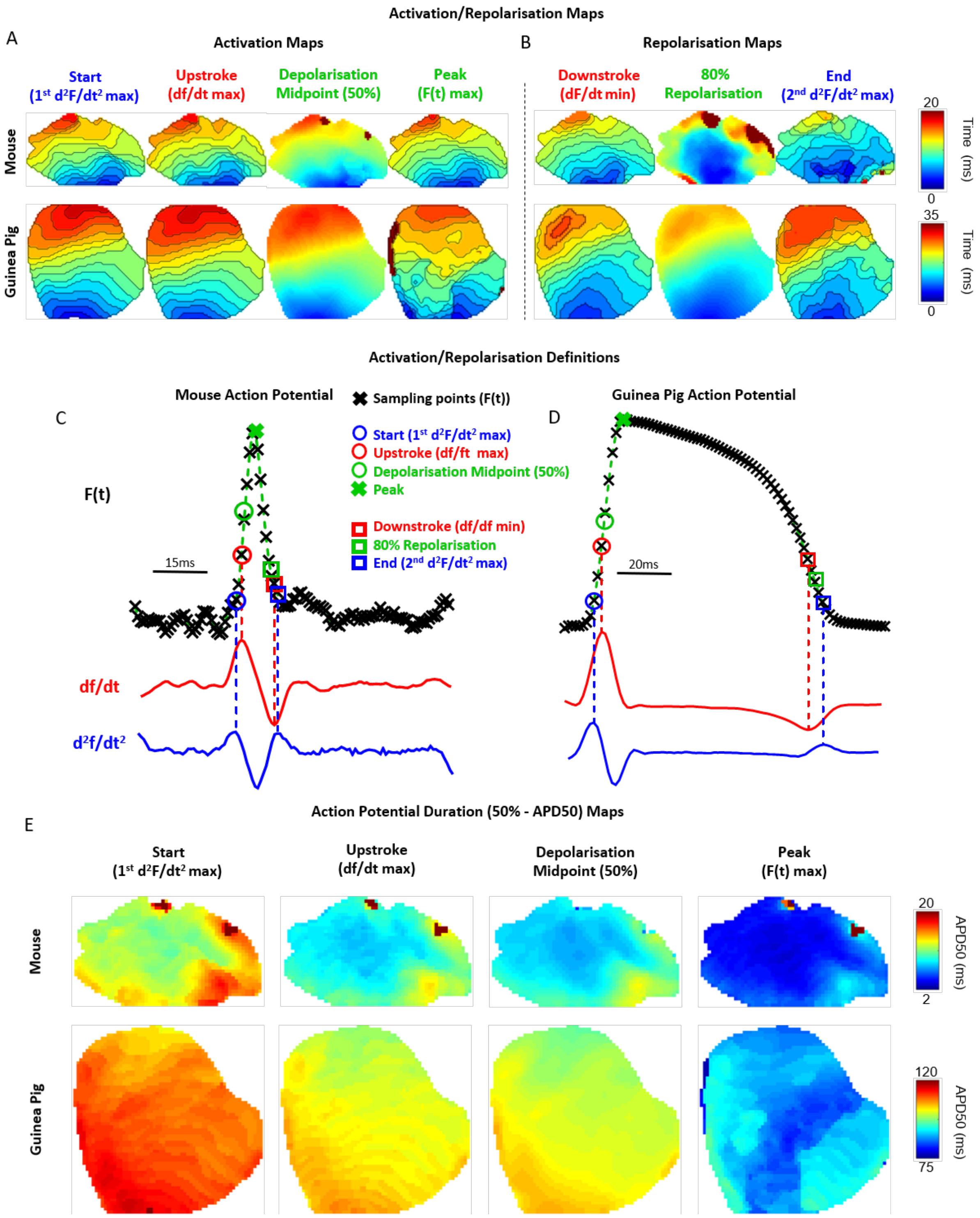

Supplementary Figure II. Activation and repolarisation definitions. A) Example activation maps from both mouse atrium and guinea pig whole heart using the four different activation definitions employable using ElectroMap. Isochronal line shown at 1ms (murine data sampling rate) and 2ms (guinea pig data sampling rate) intervals in maps where interpolation is not applied. B) Example repolarisation maps from both mouse atrium and guinea pig heart using the three different repolarisation definitions in ElectroMap. C) Optically measured murine action potential (black, F(t)) with its first (red, df/dt) and second (blue d^2F/dt^2) derivative shown below. Highlighted are the four measured activation points and three measured repolarisation points used to construct the upper activation and repolarisation maps in A&B. D) As C, but from the Guinea Pig action potential to construct the lower activation/repolarisation maps in A&B. E) Example APD50 maps from Mouse and Guinea Pig tissue

using the four different activation measures in defining start of the action potential for calculation of APD50.

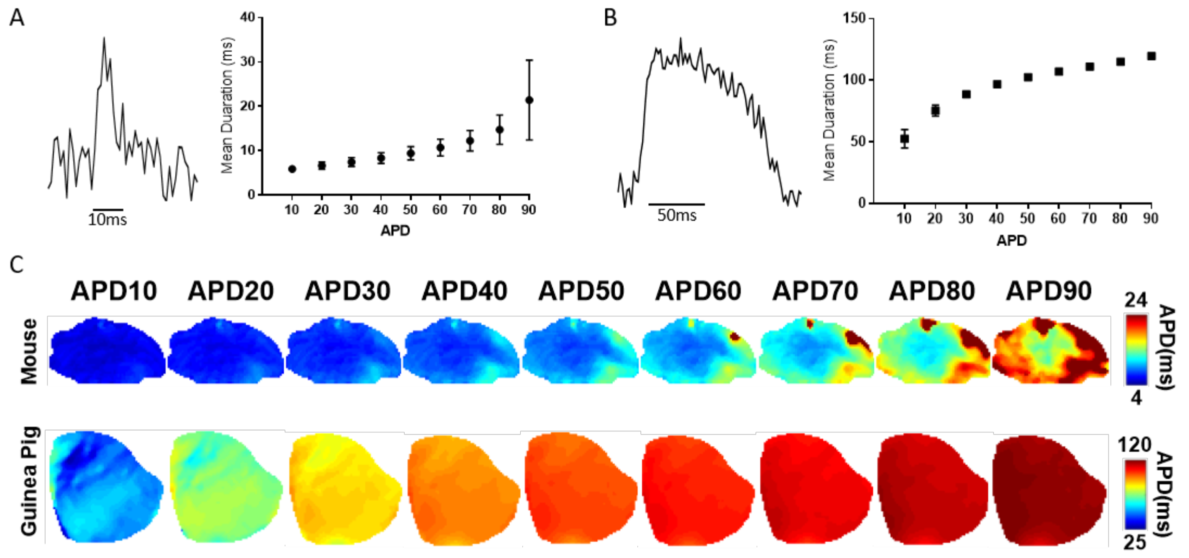

Supplementary Figure III. APD mapping. A) Example raw trace from a single pixel (left panel) and mean APD measurements as a function of user chosen repolarisation percentage in mouse atrium (right panel). B) Example raw trace from a single pixel (left panel) and mean APD measurements as a function of repolarisation percentage in guniea pig whole heart (right panel). Data shown as mean ± standard deviation. C) APD maps from murine atria and guinea pig whole heart.

;

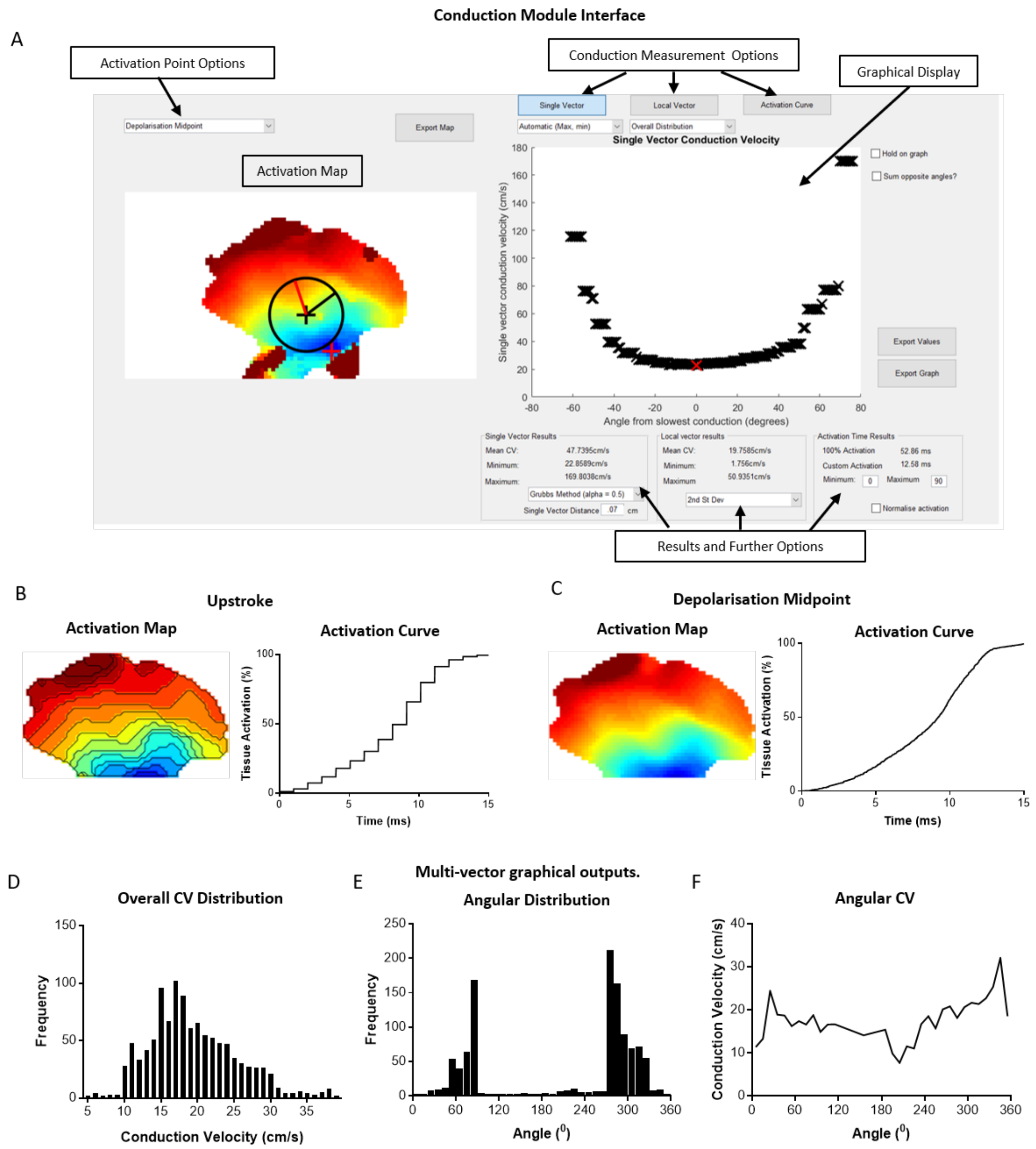

Supplementary Figure IV. Conduction Velocity Module. A) Conduction module user interface, including options for CV quantification method and activation/repolarisation definition. Here the module is shown to apply the automatic single vector method, where the user defines a distance and starting position. Possible single vector velocities are then measured at all angles in 1^0 increments from the staring position, and the results displayed in the graphical inset which plots velocity as a function of angle. This highlights the wide variation in single vector CV that can be measured from one position depending on direction. B&C) Activation maps and curves produced using two activation definitions, upstroke (B) and depolarisation midpoint (C). Maximum upstroke velocity measurement will always occur at sampling time, hence the step-wise nature of the activation curve which can make it difficult to accurately define activation constants. This is in contrast to the smooth activation curve calculated from depolarisation midpoint, as linear interpolation circumvents sampling rate limitations. D-F) Examples of the graphical plots available to the user when using the multi-vector methods. CV magnitude (E) and angular distribution (F) can be visualised as a measure of conduction heterogeneity. Equally, CV as a function of local vector angle (G) can give detailed insights into conduction pathways and mechanical structure within tissue.

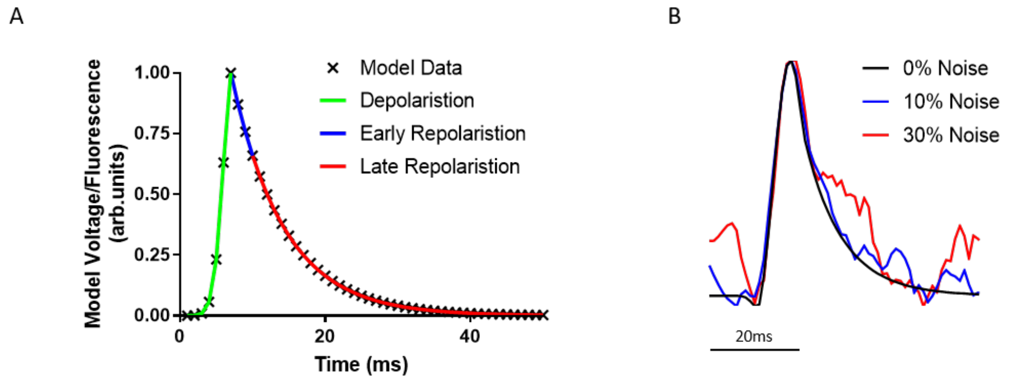

Supplementary Figure V: An example of the use of the action potential function described, black points show F(t) for t=1:50ms at 1ms intervals (i.e. sampling frequency = 1kHz). Three phases of the action potential are defined as depolarisation (green), early repolarisation (blue) and late repolarisation (red) and modelled by equations 9, 10 and 11 respectively. For activation analysis presented, only the depolarisation phase is relevant.

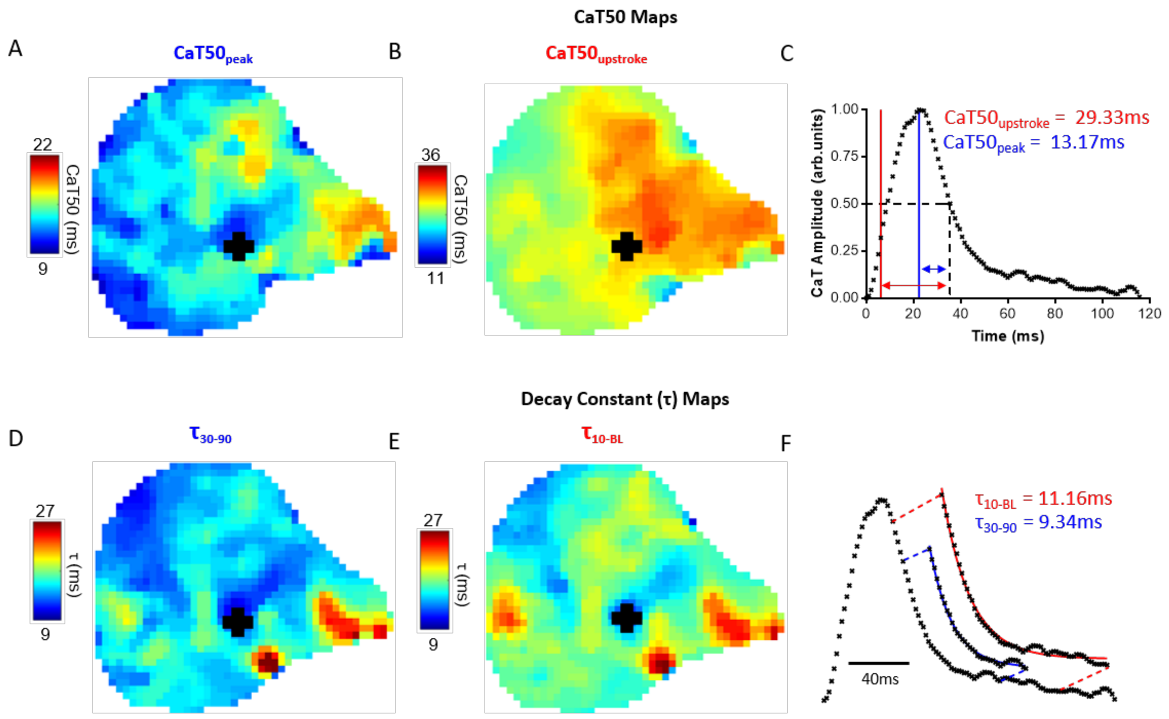

Supplementary Figure VI. Calcium analysis with variable CaT50 and decay constant settings. A) CaT50 map from murine atrium as measured from time of Ca^{2+} peak to 50% decay ($CaT50_{peak}$). B) CaT50 map from same murine atrium as measured from time of maximum upstroke velocity ($CaT50_{upstroke}$, as figure 6A). C) Example of measurement of $CaT50_{peak}$ and $CaT50_{upstroke}$ from highlighted region of interest (as figure 6A). D) Map of decay constant, τ , from same atrium. Map shows calculation of τ by fitting of exponential decay as in equation to points between 30% and 90% decay from peak (τ_{30-90} , as figure 6D). E) τ map from same atrium, but instead applying exponential decay to points between 10% decay and baseline (τ_{10-BL}). F) Example of measurement of τ_{30-90} and τ_{10-BL} from highlighted region on interest (as figure 6D).

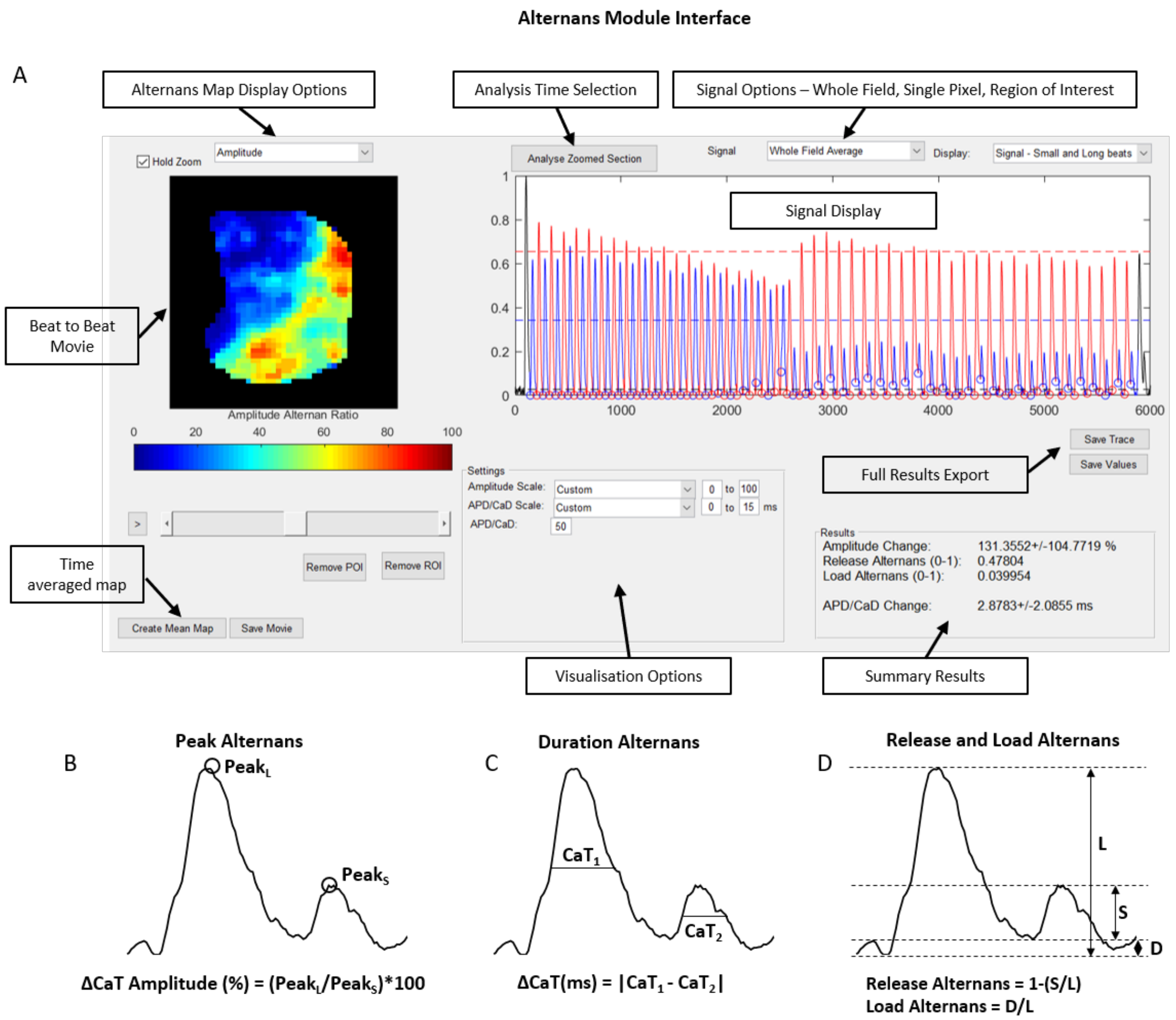

Supplementary Figure VII. Alternans Module. A) Alternans module user interface, including beat to beat display of alternans behaviour across the tissue. Analysis can be performed across whole experiment or on user defined time interval. Equally, graphical display allows analysis of whole filed average or a selected region (or single pixel) of interest. All results can easily be exported in the form of .avi/.gif for video files, a number of file formats for time-averaged images and .csv files for signal analysis. B-D) Illustrations of alternans measures used in ElectroMap applied to example Calcium signal.

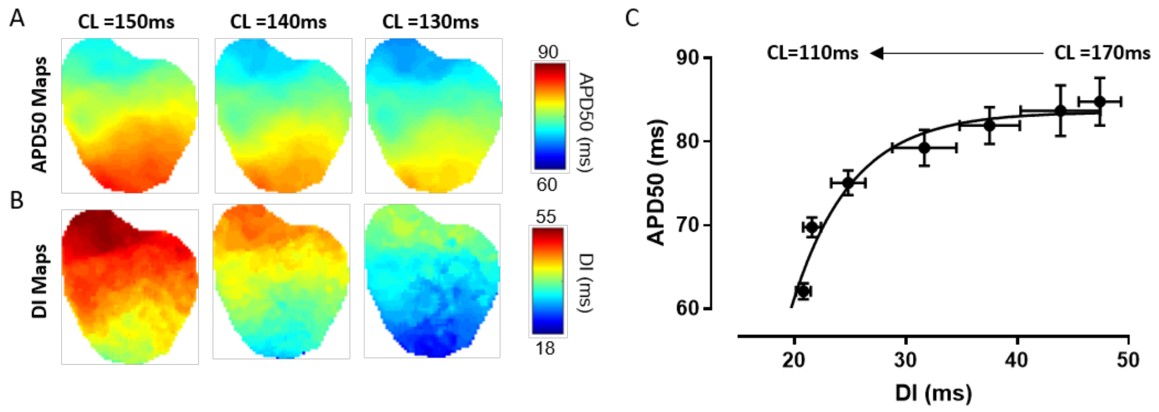

Supplementary Figure VIII. Diastolic Interval mapping. A) Example APD50 maps from guinea pig heart at decreasing cycle lengths (CL). As CL decreases (pacing frequency increases), APD50 decreases across the heart. B) Similarly, diastolic interval (DI) decreases with CL. Notably, areas of longer APD50 within the tissue concurrently show, as expected, shortened DI at all CLs. C) Mean APD50 as a function of mean DI shows that both decrease more rapidly at shorter CLs. Data shown in mean ± standard error. n=5.

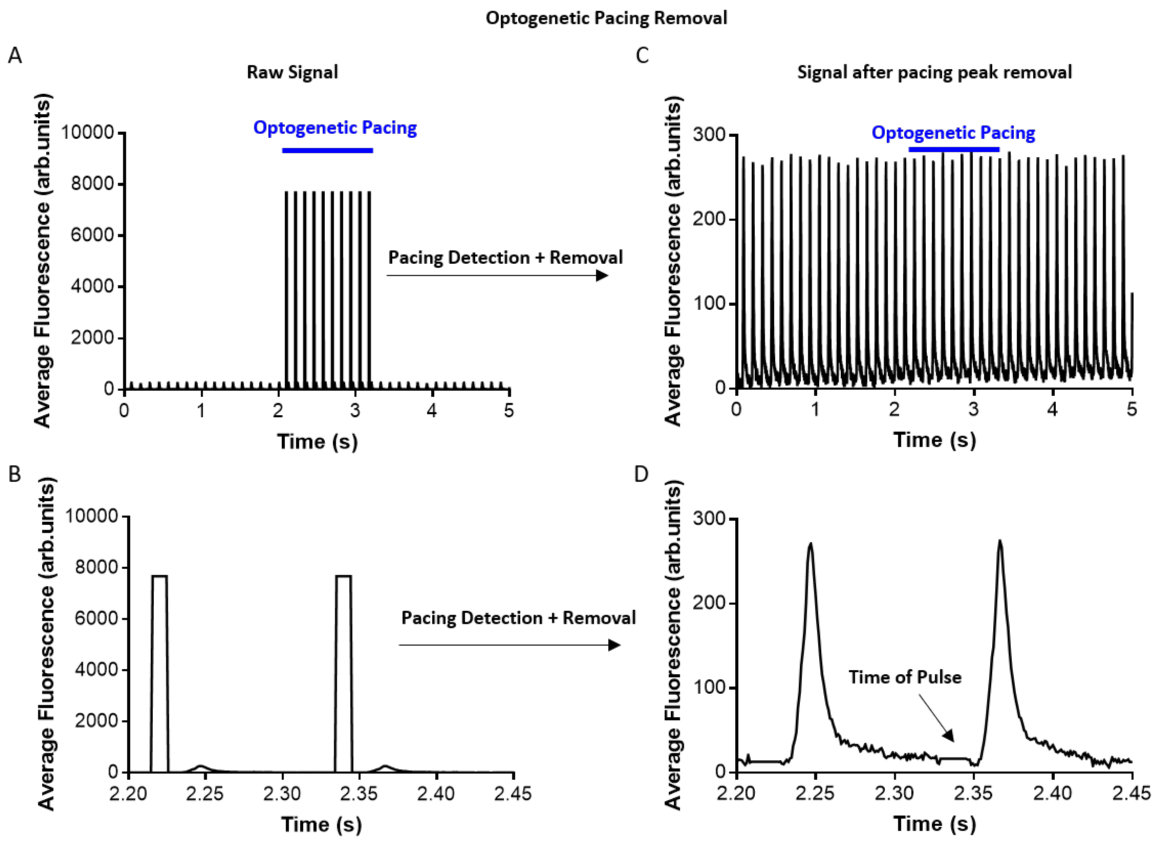

Supplementary Figure IX. Optogenetic pacing peak removal. A-B) Pseudo-Optogenetic pacing beats were added to a pre-existing dataset between 2 and 3s, as shown by the average signal. C-D) These were then automatically identified and removed using ElectroMap's 'remove frames' function.

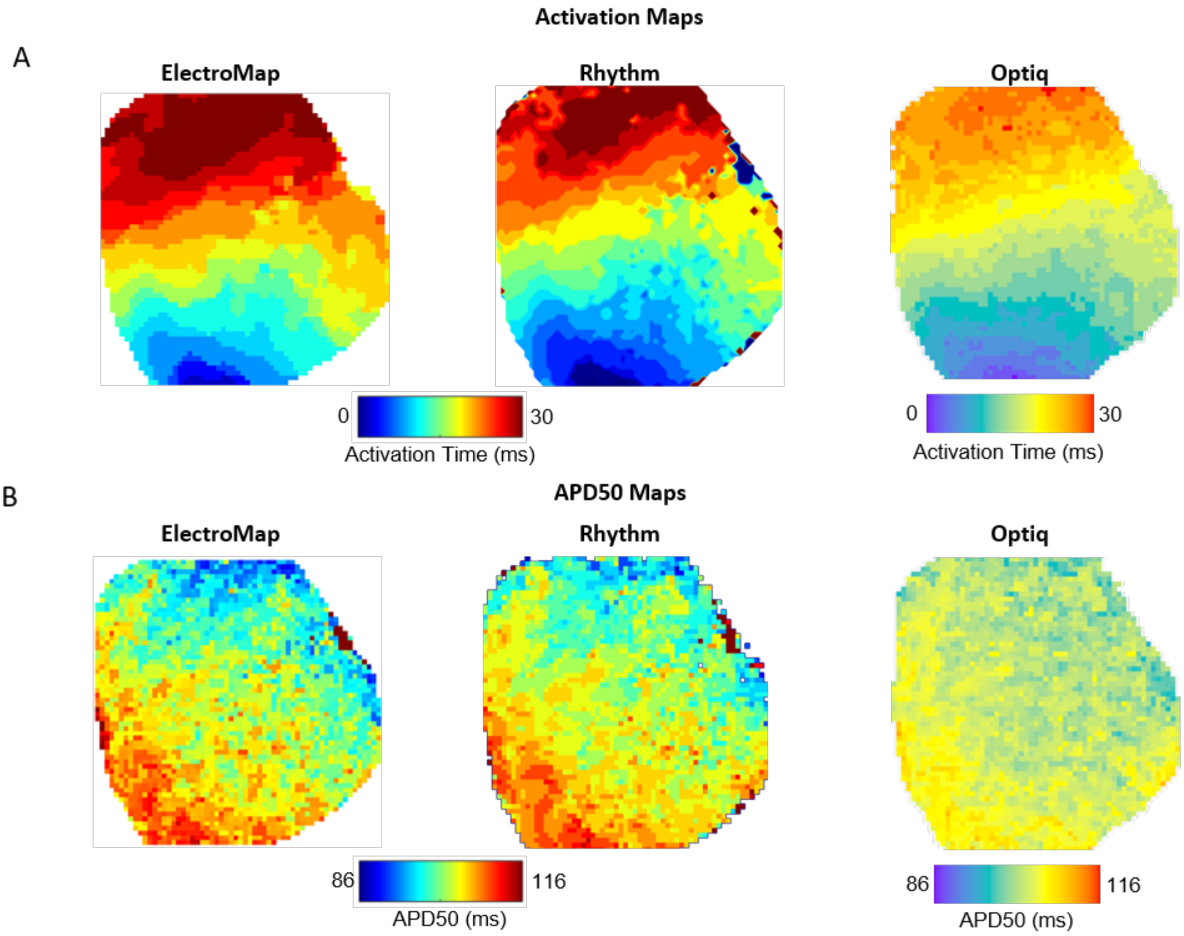

Supplementary Figure X: Software Comparison. A) Activation Map created from whole guniea pig heart using i) ElectroMap, ii) Rhythm and iii) Optiq (Cairn Research, UK). In all three cases, maximum upstroke velocity (dF/dt_{max}) was used to define time of activation. A 3x3 pixel linear ('boxcar') filter was applied to all 3 images. Other than this, no other filtering (e.g. temporal) was applied to allow direct comparison between the software. B) Whole heart APD50 maps using the same software. APD50 measured from time of maximum upstroke. The resulting mean APD50 (± standard deviation) was calculated as: ElectroMap: 102 ± 5ms, Rhythm: 103 ± 7ms, Optiq: 102 ± 3ms.

Supplemental Video Legends

Supplementary Video I: Beat-to-beat APD50 maps of guinea pig whole hearts during 70s, 5-8-5Hz experiment (Figure 5).

Supplementary Video II: Beat-to-beat APD50 maps of guinea pig whole hearts during transition from 5 to 8 Hz pacing.

Supplementary Video III: Phase mapping from virtual electrogram recordings from same patient in sinus rhythm and atrial fibrillation.

Supplemental References

1. Yu, T. Y. *et al.* An automated system using spatial oversampling for optical mapping in murine atria. Development and validation with monophasic and transmembrane action potentials. *Prog. Biophys. Mol. Biol.* **115,** 340–348 (2014).

2. Holmes, A. P. *et al.* A Regional Reduction in Ito and IKACh in the Murine Posterior Left Atrial Myocardium Is Associated with Action Potential Prolongation and Increased Ectopic Activity. *PLoS One* **11,** e0154077 (2016).

3. Syeda, F. *et al.* PITX2 Modulates Atrial Membrane Potential and the Antiarrhythmic Effects of Sodium-Channel Blockers. *J. Am. Coll. Cardiol.* **68,** 1881–1894 (2016).

4. Shattock, M. J. *et al.* Restitution slope is principally determined by steady-state action potential duration. *Cardiovasc. Res.* **113,** 817–828 (2017).

5. Winter, J. *et al.* Sympathetic nervous regulation of cardiac alternans in the intact heart. *Front. Physiol.* **9,** 1–12 (2018).

6. Krul, S. P. J. *et al.* Treatment of Atrial and Ventricular Arrhythmias Through Autonomic Modulation. *JACC Clin. Electrophysiol.* **1,** 496–508 (2015).

7. Bayly, P. V *et al.* Estimation of Conduction Velocity Vector Fields from Epicardial Mapping Data. **45,** 563–571 (1998).

8. Myles, R. C., Wang, L., Kang, C., Bers, D. M. & Ripplinger, C. M. Local β-adrenergic stimulation overcomes source-sink mismatch to generate focal arrhythmia. *Circ. Res.* **110,** 1454–1464 (2012).

9. Laughner, J. I., Ng, F. S., Sulkin, M. S., Arthur, R. M. & Efimov, I. R. Processing and analysis of cardiac optical mapping data obtained with potentiometric dyes. *AJP Hear. Circ. Physiol.* **303,** H753–H765 (2012).

10. Li, X. *et al.* An interactive platform to guide catheter ablation in human persistent atrial fibrillation using dominant frequency, organization and phase mapping. *Comput. Methods Programs Biomed.* **141,** 83–92 (2017).

11. Umapathy, K. *et al.* Phase Mapping of Cardiac Fibrillation. *Circ. Arrhythmia Electrophysiol.* **3,** 105–114 (2010).
